# Low-intensity pulsed ultrasound stimulation to treat renal fibrosis through inhibiting tubular IL-1R

**DOI:** 10.1172/jci.insight.186892

**Published:** 2025-07-29

**Authors:** Zhimin Huang, Jiaxin Dong, Ziqi Fu, Li Li, Simeng Liu, Lin Wu, Honglei Guo, Ao Bian, Kang Liu, Wei Sun, Changying Xing, Steven D Crowley, Jiafa Ren, Xiangqing Kong, Huijuan Mao

**Affiliations:** 1 Department of Nephrology and; 2 Department of Cardiology, Jiangsu Province Hospital and The First Affiliated Hospital with Nanjing Medical University, Nanjing, China.; 3 Division of Nephrology, Department of Medicine, Duke University Medical Center, Durham, North Carolina, USA.; 4 Collaborative Innovation Center for Cardiovascular Disease Translational Medicine, Nanjing Medical University, Nanjing, China.

**Keywords:** Immunology, Nephrology, Fibrosis, Therapeutics

## Abstract

Low-intensity pulsed ultrasound stimulation (LIPUS) has become increasingly appreciated for its therapeutic effect on kidney diseases. However, its role and biological mechanism in treating chronic kidney disease remain poorly defined. Here, we revealed that LIPUS was applied in a safe range with an intensity of 25–315 mW/cm^2^. Daily LIPUS at an intensity of 315 mW/cm^2^ ameliorated ischemia/reperfusion-induced (IR-induced) tubular injury and renal fibrosis, accompanied by the remarkable downregulation of IL-1R. Transcriptome sequencing showed that LIPUS significantly downregulated IL-1R and its downstream genes in IL-1β–stimulated IR-injured mice. LIPUS effectively reversed IL-1β–induced tubular injury and reduced the production of pro-fibrotic cytokines by downregulating IL-1R in vivo and in vitro. Renal proximal tubule–specific *Il1r1*-knockout mice exhibited milder renal tubular injury and fibrosis after IR injury. However, LIPUS did not ameliorate IR injury in proximal tubule–specific *Il1r1*-knockout mice. Collectively, daily LIPUS at an intensity of 315 mW/cm^2^ relieves IR-induced tubular injury and fibrosis, potentially by downregulating tubular IL-1R.

## Abstract 2

LIPUS relieves IR-induced tubular injury and fibrosis by down-regulating tubular IL-1R, and daily LIPUS at an intensity of 315 mW/cm^2^ may be a promising treatment for CKD.

## Graphical abstract

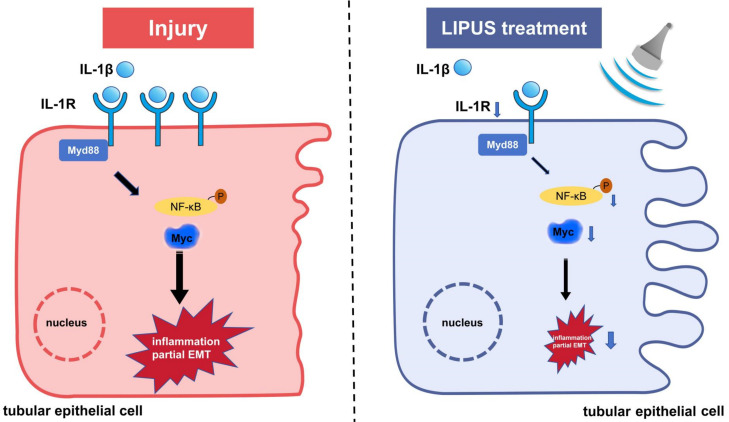

## Introduction

Chronic kidney disease (CKD) and the substantial socioeconomic burden of CKD-induced renal dysfunction constitute a serious public health concern (1, 2). Renal fibrosis is a common outcome of CKD characterized by varying degrees of extracellular matrix deposition in the renal tissue, which eventually leads to end-stage renal failure (3, 4). Tubular epithelial cells experience a partial epithelial-mesenchymal transition (EMT) during renal fibrosis. Therefore, inhibiting the partial EMT in tubular epithelial cells has been suggested as a potential antifibrosis strategy (5, 6).

Noninvasive ultrasound may realize this therapeutic strategy. Low-intensity pulsed ultrasound stimulation (LIPUS) is an ultrasound technology that delivers low-intensity and pulsed waves (7). LIPUS has emerged as a versatile therapeutic modality in diverse clinical applications, including fracture healing, neuromodulation, treatment of myocardial fibrosis, and various kidney diseases (7–11). The parameters in the protocol are typically set as follows: ultrasound intensities of 30–500 mW/cm² delivered at frequencies ranging from 0.5–1.5 MHz, pulse repetition frequencies of 100–1,000 Hz, and duty cycles of 1%–20% (7–9). However, the biological mechanism underlying the therapeutic role of LIPUS remains largely unclear. Herein, we analyzed how LIPUS relieves renal tubular injury and fibrosis by managing IL-1β/IL-1R, aiming to provide guidance for the clinical management of CKD via LIPUS.

## Results

### Effect of LIPUS on mouse tubular epithelial cells and screening of effective LIPUS parameters in vitro.

**Figure 1:**
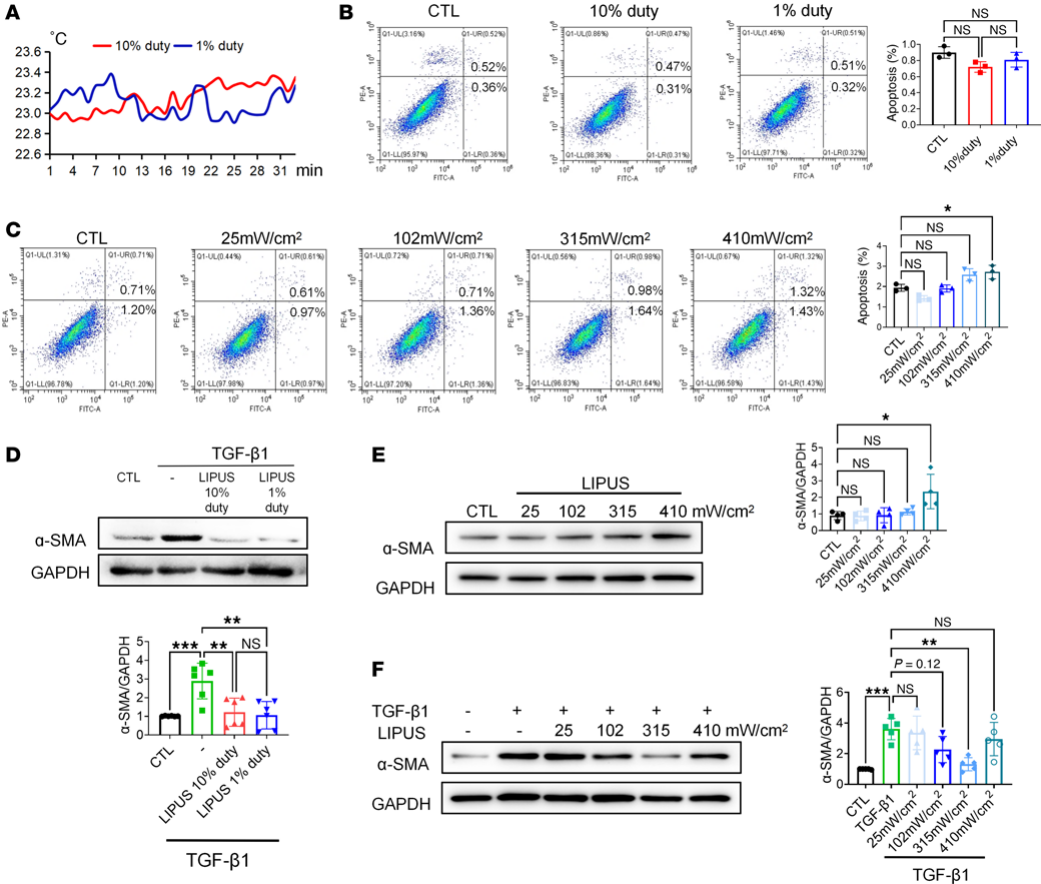
In vitro effect of LIPUS on MTECs and parameter selection for CKD treatment. (**A**) The thermal effect of LIPUS with different duty cycles on MTECs. (**B** and **C**) Flow cytometry of apoptosis of MTECs treated with LIPUS at varying duty cycles (**B**) and intensities (**C**) and quantification of apoptotic cells (Q2 + Q3). (*n* = 3). (**D**) Protein levels of α-SMA in MTECs induced with 15 ng/mL TGF-β1 (*n* = 6). (**E**) Protein levels of α-SMA in MTECs treated with LIPUS at varying intensities (*n* = 4). (**F**) Protein levels of α-SMA in MTECs treated with LIPUS at varying intensities stimulated by 15 ng/mL TGF-β1 (*n* = 5). Data are presented as mean ± SD and were analyzed using 1-way ANOVA with Tukey’s post hoc test. *P < 0.05, **P < 0.01, ***P < 0.001.

LIPUS is a therapeutic technology that utilizes pulsed acoustic waves to treat inflammatory conditions, such as acute kidney injury (AKI) and CKD (8, 9). For the treatment of kidney diseases, the maximum LIPUS intensity reported in the literature is 100 mW/cm² (8, 10, 11). In our study, LIPUS at an intensity of 102 mW/cm^2^ with 10% or 1% duty cycle provided a minimal thermal effect on mouse tubular epithelial cells (MTECs) (Figure 1A). Flow cytometry was applied to detect the apoptosis of MTECs stimulated by LIPUS with varying duty cycles (Figure 1B). The results showed that the apoptotic rate was comparable in MTECs treated by LIPUS with either 10% or 1% duty cycle (*P* > 0.05). To determine the optimal duty cycle, MTECs were treated with TGF-β1. Interestingly, both 10% and 1% duty cycles of LIPUS showed similar efficacy in partially reversing the TGF-β1–induced overexpression of the fibrotic marker α–smooth muscle actin (α-SMA) in MTECs (Figure 1D). Overall, 1% duty cycle was adopted in further studies.

We later assessed the best performing intensity of LIPUS on MTECs. The apoptotic rates were similar in MTECs treated with LIPUS at intensities of 25, 102, and 315 mW/cm^2^. However, LIPUS at an intensity of 410 mW/cm^2^ resulted in a higher apoptotic rate and expression level of α-SMA compared with the untreated group (Figure 1, C and E). In addition, TGF-β1–induced overexpression of α-SMA was not reversed by 410 mW/cm^2^ LIPUS but partially by LIPUS at an intensity of 315 mW/cm^2^ (Figure 1F). Collectively, LIPUS at an intensity of less than 315 mW/cm^2^ is safe to MTECs.

Considering the LIPUS transducer was placed over the skin of the back on both kidneys, and podocytes play a fundamental role in the glomerular filtration barrier (12), the safety of LIPUS on podocytes was investigated. No significant differences in the apoptotic rate and expression level of podocin (podocyte marker) were detected between podocytes treated with LIPUS at varying intensities or not (Supplemental Figure 1; supplemental material available online with this article; https://doi.org/10.1172/jci.insight.186892DS1), suggesting the safety of LIPUS at less than 600 mW/cm^2^ for podocytes.

Overall, we consistently applied LIPUS at fixed parameters (315 mW/cm² intensity, 1% duty cycle) throughout all subsequent in vivo and in vitro experiments.

### Frequency of LIPUS in vivo.

**Figure 2:**
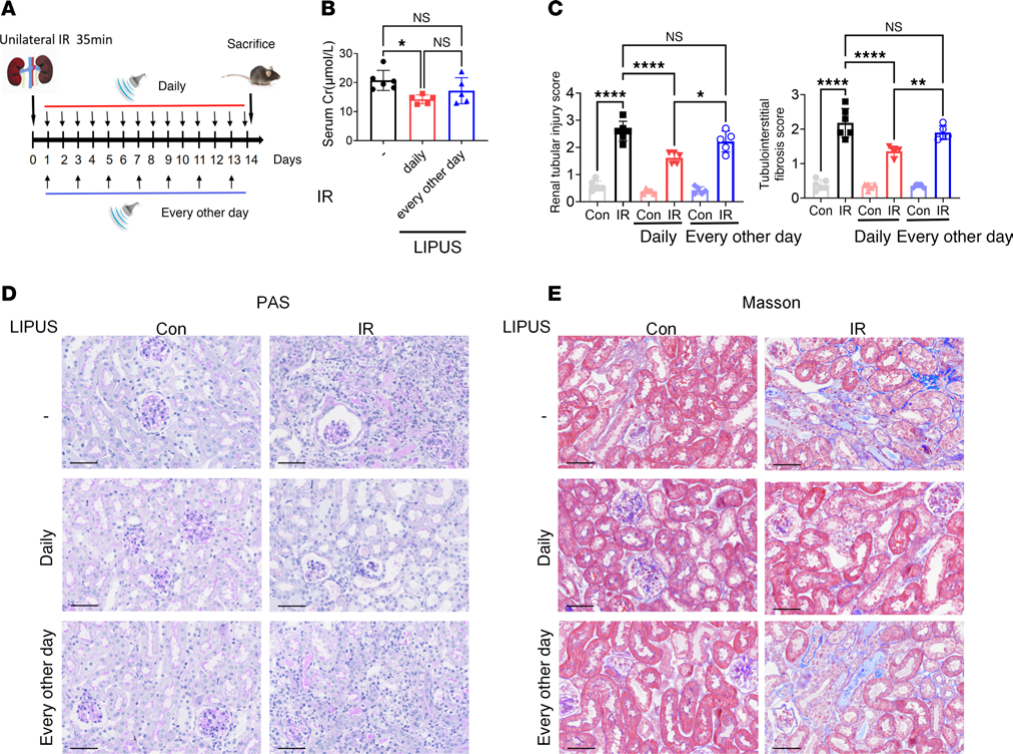
LIPUS parameter evaluation for IR-induced renal injury. (**A**) Study protocol. (**B**) Serum creatinine (Cr, a blood biomarker of kidney function) levels in IR-induced mice with LIPUS. (C) Tubular injury and tubulointerstitial fibrosis scores. (D and E) Representative microscopic images showing PAS staining (D) and Masson’s trichrome staining (E) of tubular injury and collagen deposition in renal tissue. Scale bar: 50 μm. Data presented as mean ± SD. Statistical analysis was performed using 1-way ANOVA followed by Tukey’s test. *P < 0.05, **P < 0.01, ****P < 0.0001. n = 6 in contralateral (Con) or IR-injured kidneys; n = 5 in Con or IR-injured kidneys with every-other-day LIPUS treatment; n = 5 in Con or IR-injured kidneys with daily LIPUS treatment.

Two weeks after renal ischemia/reperfusion (IR) induction in mice, increased serum creatinine (Cr, a blood biomarker of kidney function) and histological abnormalities in the kidney suggested the typical chronic unilateral kidney damage of CKD (13). Specifically, detached brush border, necrotic tubular epithelial cells, various tubular casts, massive tubulointerstitial mononuclear cell infiltration, and accumulated deposition of collagen in the mouse kidney were visualized by Periodic Acid–Schiff (PAS) and Masson’s trichrome staining. To investigate the in vivo therapeutic efficacy of LIPUS on CKD mice, a 14-day treatment regimen was performed using a 1 MHz transducer at an intensity of 315 mW/cm². LIPUS was administered to bilateral kidneys for 20 minutes per kidney daily or every other day, starting from the day after IR surgery (Figure 2A).

Daily LIPUS improved the renal function compared with the diseased group (Figure 2B). Histologically, the tubular injury and renal fibrosis scores in CKD mice treated with daily LIPUS decreased significantly (Figure 2, C–E). However, serum Cr levels in CKD mice were not influenced by the frequency of LIPUS, which may be attributed to the preservation of the contralateral kidney (*P* > 0.05, Figure 2B). A mild histological renal injury, probably caused by circulating factors associated with renal injury, was observed in the contralateral kidney. In the following experiments, mice in the sham operation group were considered controls.

The safety of LIPUS was evaluated using both systemic and local tissue assessments. Hematological analysis demonstrated that blood parameters were preserved across all groups, with comparable hemoglobin concentrations, leukocyte counts, and platelet counts (Supplemental Figure 2A). Hepatic function markers, including alanine aminotransferase (ALT) and aspartate aminotransferase (AST), remained within normal ranges without intergroup differences (all *P* > 0.05) (Supplemental Figure 2B). Histological evaluation of transducer-contacted skin and hepatic parenchyma revealed normal tissue architecture, with no evidence of inflammatory infiltration, cellular necrosis, or other pathological changes in H&E-stained sections (Supplemental Figure 2C). These data at systemic and tissue levels verify the safety of our optimized LIPUS protocol.

Taken together, based on our findings, we determined that daily LIPUS would be used to treat CKD mice in vivo.

### LIPUS improves renal function and ameliorates IR-induced renal inflammation and tubular injury.

**Figure 3:**
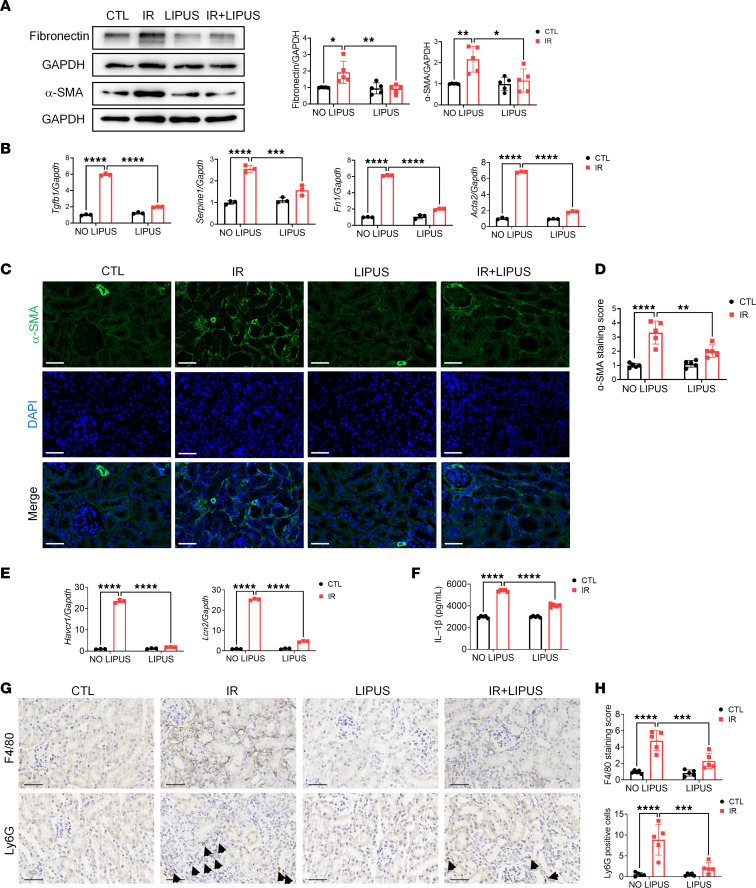
LIPUS protects from IR-induced renal injury. (**A**) Protein levels of fibronectin and α-SMA in IR-induced renal tissue of LIPUS-treated or untreated mice and the quantitative analyses. (**B**) The mRNA expressions of *Tgfb1*, *Serpine1*, *Fn1*, and *Acta2*. (**C**) Immunofluorescence staining of α-SMA (green) and DAPI/DNA (blue) in renal tissue. Scale bar: 50 μm. (**D**) Quantitative results of positive staining of α-SMA. (**E**) The mRNA expressions of *Havcr1* and *Lcn2* in renal tissue. (**F**) IL-1β level in renal tissue detected by ELISA. (**G**) Immunohistochemical staining of F4/80 and Ly6G in renal tissue. Scale bar: 50 μm. (**H**) Quantitative results of positive staining of F4/80 and Ly6G. Data information: Data are presented as mean ± SD. Statistical significance was determined by 2-way ANOVA followed by post hoc Šídák’s multiple comparisons test. *P < 0.05, **P < 0.01, ***P < 0.001, ****P < 0.0001. *n* = 5 in each group.

Fibronectin and α-SMA were significantly downregulated in renal tissue of LIPUS-treated CKD mice, compared with those not treated (Figure 3A). LIPUS drastically downregulated the mRNA levels of renal fibrosis markers, such as *Tgfb1*, *Serpine1*, *Fn1*, and *Acta2* (Figure 3B). Immunofluorescence staining further supported the antifibrotic efficacy of LIPUS in suppressing the overexpressed α-SMA around renal tubular cells (Figure 3, C and D). Additionally, LIPUS significantly downregulated the mRNA levels of renal tubular injury markers, including *Havcr1* and *Lcn2*, in renal tissue of CKD mice compared with those not treated (Figure 3E). Collectively, LIPUS effectively protected against renal fibrosis and tubular injury in CKD mice.

Inflammation is a critical pathogenic characteristic of renal fibrosis (14). The number of renal macrophages was much higher in CKD mice than in controls (Figure 3G) and remarkably reduced by 51.88% after LIPUS (Figure 3, G and H). Consistently, IR-induced tubulointerstitial neutrophil infiltration was dramatically abrogated by LIPUS (Figure 3, G and H). ELISA data revealed that the increase in IL-1β level in renal tissue of CKD mice was significantly reversed by LIPUS (Figure 3F).

To directly exclude the potential compensatory effects of the contralateral kidney on our data, we validated our results using experiments in which the contralateral kidneys were surgically removed 14 days after unilateral IR injury. At 24 hours after nephrectomy, serum Cr levels were significantly lower in the LIPUS-treated group (mean ± SD 28.09 ± 2.14 μmol/L), compared with the CKD group (41.86 ± 6.09 μmol/L, *P* < 0.01), demonstrating a 33% improvement in renal function that can be uniquely attributed to the protective effects of LIPUS on the injured kidney, independent of contralateral compensation (Supplemental Figure 3).

Together, LIPUS was an effective approach to treat renal fibrosis, tubular injury, and inflammatory response in CKD mice.

### LIPUS downregulates IL-1R and its downstream genes.

**Figure 4:**
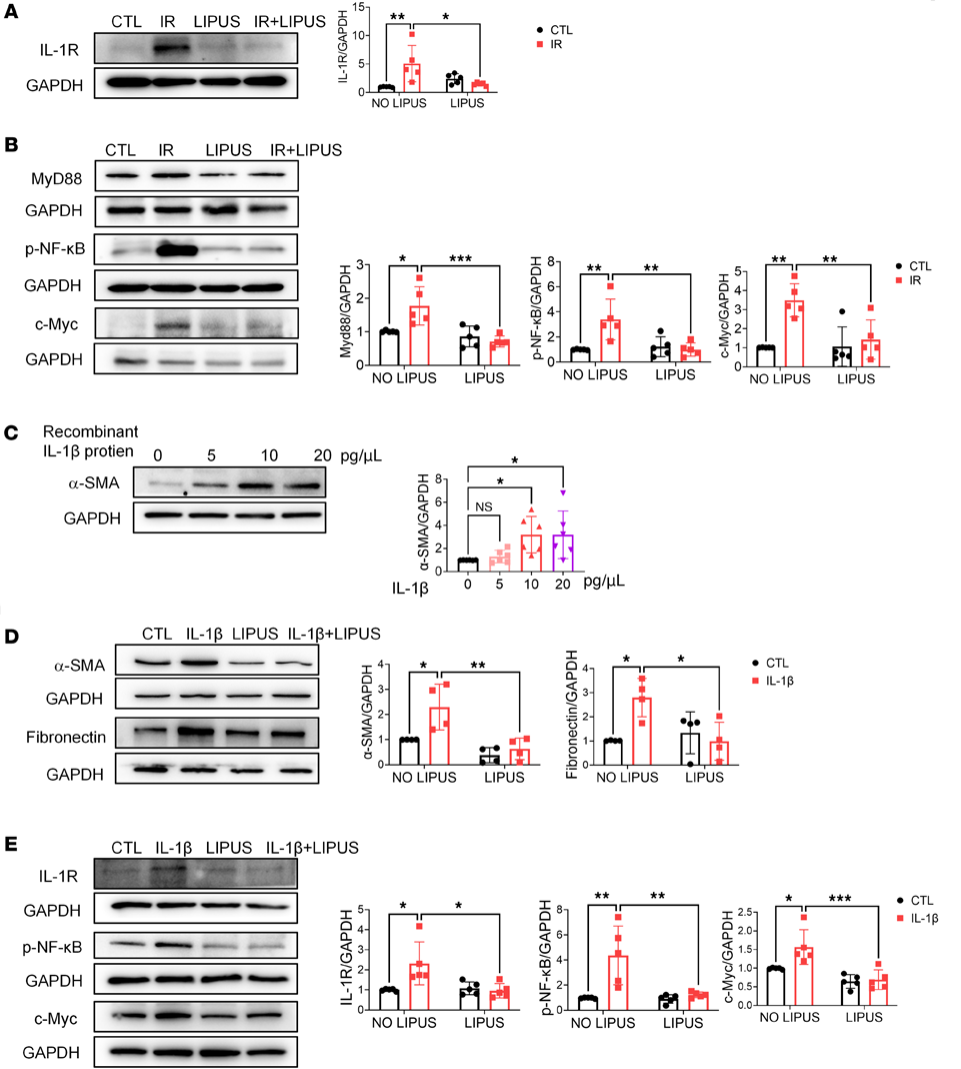
LIPUS downregulates IL-1R and its downstream genes. (**A**) Protein level of IL-1R in IR-induced renal tissue and its quantitative analysis. (*n* = 5 in each group.) (**B**) Protein levels of MyD88, p-NF-κB, and c-Myc in IR-induced renal tissue and their quantitative analyses (*n* = 5). (**C**) Protein level of α-SMA in MTECs induced by different IL-1β concentrations and the quantitative analysis (*n* = 6). (**D**) Protein levels of α-SMA and fibronectin in 10 pg/μL IL-1β–induced MTECs and the quantitative analyses (*n* = 4). (**E**) Protein levels of IL-1R, p-NF-κB, and c-Myc in 10 pg/μL IL-1β–induced MTECs and the quantitative analyses. (*n* = 5). Data information: Data are presented as mean ± SD. Statistical significance was determined by 1-way ANOVA with Tukey’s test (**C**) and 2-way ANOVA with Šídák’s multiple comparisons test (**A**, **B**, **D**, and **E**). *P < 0.05, **P < 0.01, ***P < 0.001.

Increasing evidence has validated the role of IL-1β in kidney diseases. IL-1β and its activation induce proximal tubule cell atrophy and interstitial collagen deposition (15, 16). Through binding to the cell surface receptor IL-1R, IL-1β triggers the activation of pro-inflammatory signaling pathways within cells. IL-1R further activates the NF-κB and c-Myc transcription factors through myeloid differentiation primary response protein 88 (MyD88) (17, 18). Searching in the publicly available database of kidney interactive transcriptomics (http://humphreyslab.com/SingleCell/), we found *Il1b* was overexpressed in macrophages (Supplemental Figure 4A), and *Il1b* was significantly downregulated in LIPUS-treated renal tissue of CKD mice (Figure 3F). Additionally, the genes encoding *Il1r1* in the damaged proximal tubules were activated in both IR-induced and unilateral ureteral obstruction–induced (UUO-induced) CKD models (Supplemental Figure 4B). Moreover, overexpressed IL-1R in renal tissue of CKD mice was significantly downregulated by LIPUS (Figure 4A).

The regulatory effect of LIPUS on IL-1R downstream proteins was then investigated. In vivo, LIPUS significantly downregulated IL-1R downstream proteins MyD88, phosphorylated (p-) NF-κB, and c-Myc, which were initially overexpressed in diseased renal tissue of CKD mice (Figure 4B). In vitro, IL-1β induction caused a remarkable overexpression of α-SMA in MTECs (Figure 4C). Surprisingly, LIPUS not only reduced IL-1β–induced partial tubular EMT but also reversed IL-1R overproduction within MTECs. Moreover, LIPUS silenced the IL-1β–induced NF-κB signaling pathway. Overexpression of c-Myc in IL-1β–induced MTECs was consistently suppressed by LIPUS (Figure 4, D and E). Collectively, LIPUS inhibited IL-1R and its downstream proteins in CKD.

### LIPUS protects IR-injured mice stimulated by IL-1β and downregulates IL-1R.

**Figure 5:**
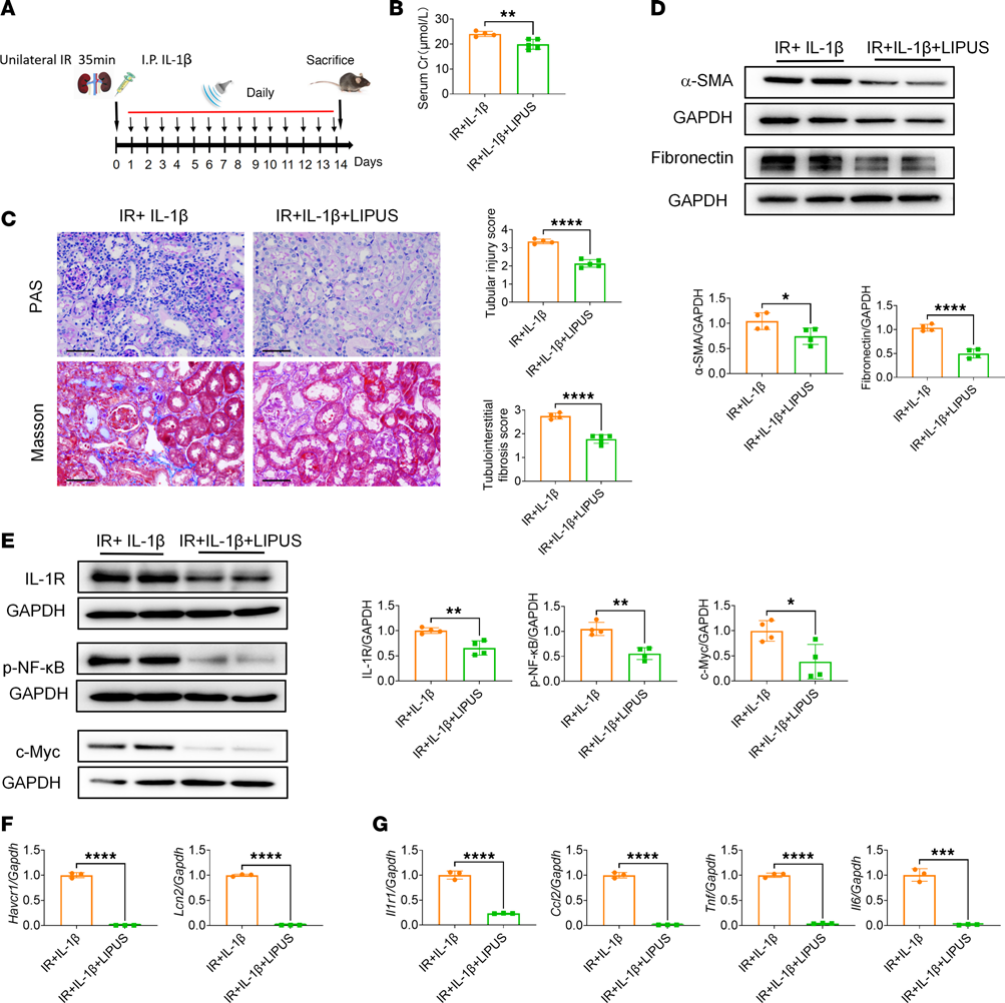
LIPUS ameliorates IL-1β–stimulated IR-induced renal injury. (**A**) Study protocol. (**B**) Serum Cr levels. (*n* = 4 in IL-1β-stimulated IR-induced renal injury group without treatment; IR+IL-1β. *n* = 5 in IL-1β–stimulated IR-induced renal injury group with LIPUS treatment; IR+IL-1β+LIPUS.) (**C**) Tubular injury and tubulointerstitial fibrosis scores and representative pathological visualizations. Scale bar: 50 μm. (*n* = 4–5.) (**D**) Protein levels of α-SMA and fibronectin in IL-1β–stimulated IR-induced renal injury with or without LIPUS treatment and the quantitative analyses (*n* = 4). (**E**) Protein levels of IL-1R, p-NF-κB, and c-Myc in IL-1β–stimulated IR-induced renal injury with or without LIPUS treatment, and the quantitative analyses (*n* = 4). (**F**) The mRNA expressions of *Havcr1* and *Lcn2* in IL-1β–stimulated IR-induced renal injury with or without LIPUS treatment (*n* = 3). (**G**) The mRNA expressions of *Il1r1*, *Ccl2*, *Tnf*, and *Il6* in IL-1β–stimulated IR-induced renal injury with or without LIPUS treatment (*n* = 3). Data information: Data are presented as mean ± SD. Statistical analysis was performed using Student’s *t* test. *P < 0.05, **P < 0.01, ***P < 0.001, ****P < 0.0001.

To further elucidate the role of IL-1R in the therapeutic efficacy of LIPUS on renal IR injury, IR-injured mice were exposed to IL-1β stimulation for 2 weeks, in the presence or absence of LIPUS. In the absence of LIPUS treatment, renal function was significantly worse in IR-injured mice stimulated by IL-1β than in those with LIPUS intervention (Figure 5, A and B). Consistently, the tubular injury and renal fibrosis scores were lower in LIPUS-treated CKD mice with IL-1β stimulation, compared with those in diseased renal tissue without LIPUS treatment (Figure 5C). Expression levels of fibrotic markers (fibronectin and α-SMA), IL-1R downstream proteins (IL-1R, p-NF-κB, and c-Myc), tubular injury markers (*Havcr1* and *Lcn2*), and inflammatory factors (*Ccl2*, *Tnf*, and *Il6*) were also significantly decreased in IL-1β–stimulated renal tissue with LIPUS treatment in comparison with untreated diseased renal tissue (Figure 5, D–G).

**Figure 6:**
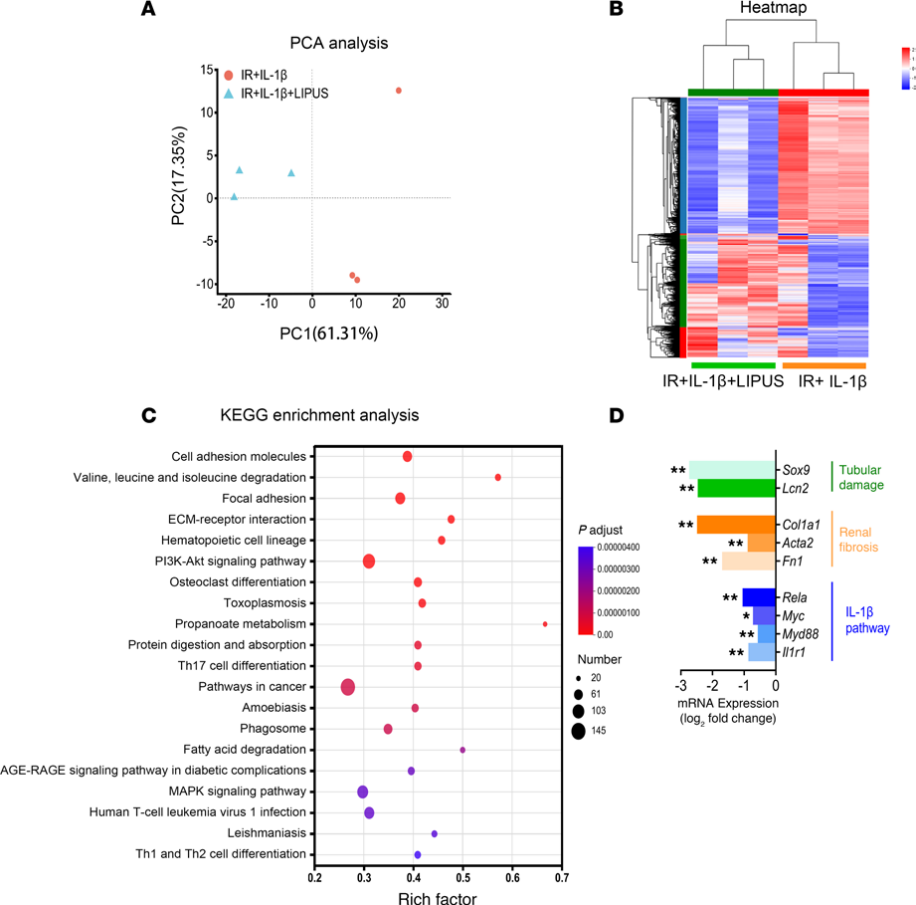
RNA sequencing of IL-1β–stimulated IR-induced renal tissue with or without LIPUS. (**A**) Two-dimensional PCA representation of kidney transcriptomes at day 14. (**B**) A heatmap visualizing DEGs between LIPUS-treated and untreated groups (*n* = 3). (**C**) KEGG enrichment analysis of DEGs. The top 20 significantly enriched pathways are listed. (**D**) Expression levels of IL-1R and its downstream genes, renal fibrosis markers, and tubular damage markers. Data information: *P value < 0.05, **P adjust value < 0.05. PCA, principal component analysis; DEGs, differentially expressed genes; KEGG, Kyoto Encyclopedia of Genes and Genomes.

The above results demonstrated that IL-IR might be essential for the efficacy of LIPUS on CKD mice stimulated by IL-1β. To unveil the molecular mechanisms underlying the involvement of IL-1R in LIPUS treatment of CKD, we searched the differentially expressed genes (DEGs) in the IR-induced renal tissue of mice with IL-1β stimulation between the LIPUS treatment group and the nontreatment group. Visualized in a heatmap, the principal component analysis (PCA) showed 3,570 DEGs, including 1,702 upregulated and 1,868 downregulated genes (Figure 6, A and B). Kyoto Encyclopedia of Genes and Genomes (KEGG) enrichment analysis identified the main biochemical metabolic pathways enriched in DEGs. Among them, the MAPK signaling pathway and fatty acid degradation, which are associated with IL-1R activation in renal tubules (19), were significantly enriched (Figure 6C). Notably, genes encoding the IL-1R pathway, fibrotic markers, tubular injury markers, regulator of partial EMT in renal tubules (e.g., *Sox9*, 0.15-fold downregulation, *P* = 0.018, Figure 6D) (6), and regulators of fatty acid metabolism (e.g., *Apom*, 3.06-fold upregulation, *P* = 3.53 × 10^–8^) were also differentially expressed (19). Together, the tubular IL-1R signaling pathway was predicted as the underlying mechanism explaining the therapeutic effect of LIPUS on CKD.

### LIPUS treats CKD by regulating the tubular IL-1R signaling pathway.

**Figure 7:**
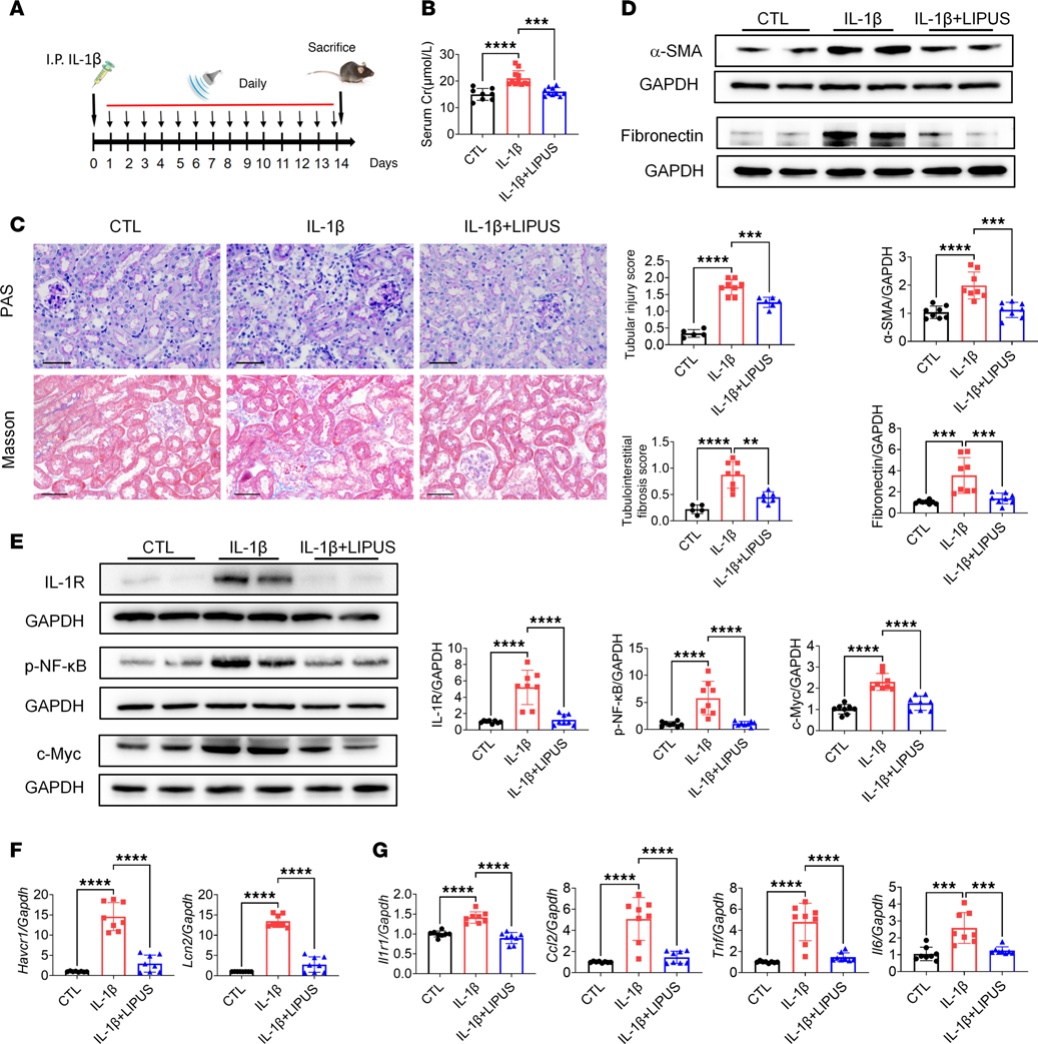
LIPUS protects from IL-1β–induced renal injury. (**A**) Study protocol. (**B**) Serum Cr levels (*n* = 8 in control group, *n* = 11 in IL-1β stimulation group, *n* = 10 in IL-1β-induced renal injury group with LIPUS treatment; IL-1β+LIPUS). (**C**) Tubular injury and tubulointerstitial fibrosis scores and representative pathological visualizations. Scale bar: 50 μm. (*n* = 5–8.) (**D**) Protein levels of α-SMA and fibronectin in renal tissues and the quantitative analyses (*n* = 8). (**E**) Protein levels of IL-1R, p-NF-κB, and c-Myc in renal tissues and the quantitative analyses (*n* = 8). (**F**) The mRNA expressions of *Havcr1* and *Lcn2* in renal tissues (*n* = 8). (**G**) The mRNA expressions of *Il1r1*, *Ccl2*, *Tnf*, and *Il6* in renal tissues (*n* = 8). Data information: Data are presented as mean ± SD, with significance determined by 1-way ANOVA with Tukey’s post hoc test. **P < 0.01, ***P < 0.001, ****P < 0.0001.

To further verify the role of IL-1R in the therapeutic efficacy of LIPUS on renal fibrosis and tubular injury, we subjected mice to IL-1β stimulation and LIPUS (Figure 7A). Compared with levels in IL-1β–stimulated mice without LIPUS treatment, serum Cr levels were significantly lower in LIPUS-treated mice following IL-1β stimulation (Figure 7B). LIPUS partially reversed pathological injuries in IL-1β–stimulated mice, including tubular atrophy, cast formation, and interstitial matrix deposition (Figure 7C and Supplemental Figure 5). Moreover, levels of fibrotic markers (fibronectin and α-SMA), IL-1R and its downstream proteins (p-NF-κB and c-Myc), tubular injury markers (*Havcr1* and *Lcn2*), and inflammatory factors (*Ccl2*, *Tnf*, and *Il6*) were inhibited by LIPUS treatment (Figure 7, D–G).

**Figure 8:**
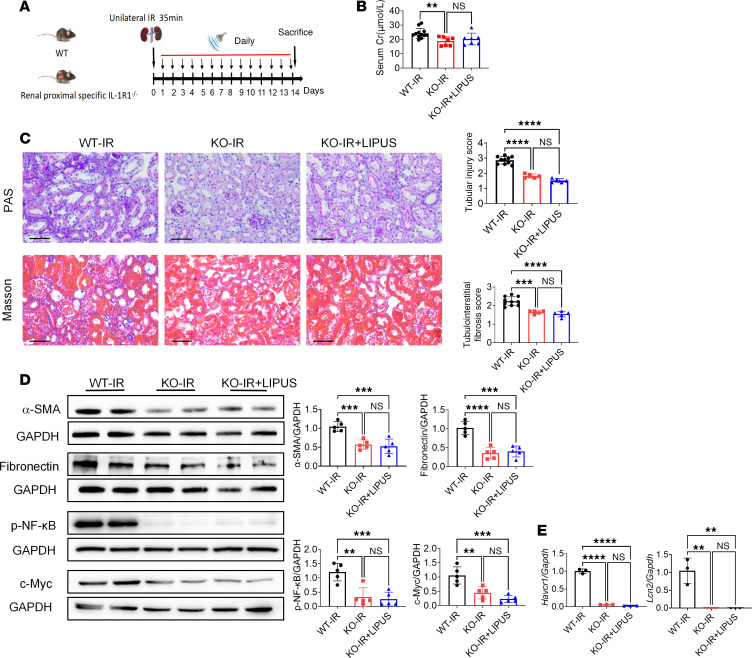
LIPUS treats CKD by regulating the tubular IL-1R signaling pathway. (**A**) Study protocol. (**B**) Serum Cr levels in *Pepck-Cre^+^ Il1r1*^fl/fl^ (KO) mice. (*n* = 12 in WT-IR group of *Pepck-Cre^–^ Il1r1*^fl/fl^ mice, and *n* = 7 in other groups of *Pepck-Cre^+^ Il1r1*^fl/fl^ mice.) (**C**) Tubular injury and tubulointerstitial fibrosis scores in *Pepck-Cre^+^ Il1r1*^fl/fl^ mice and representative pathological visualizations. Scale bar: 50 μm. (*n* = 5–10.) (**D**) Protein levels of α-SMA, fibronectin, p-NF-κB, and c-Myc in renal tissues and the quantitative analyses (*n* = 5). (**E**) The mRNA expressions of *Havcr1* and *Lcn2* in renal tissues (*n* = 3). Data information: Data are presented as mean ± SD, with significance determined by 1-way ANOVA with Tukey’s post hoc test. **P < 0.01, ***P < 0.001, ****P < 0.0001.

Based on the above findings, we speculated that the role of LIPUS in inhibiting renal tubular injury and fibrosis was attributed to IL-1R. Regulatory effects of IL-1 on tubular epithelial cells are mediated through IL-1R (20). To evaluate the function of tubular IL-1R in suppressing renal fibrosis by IL-1, we prepared renal proximal tubule–specific *Il1r1*-knockout mice. Following IR injury, *Pepck-Cre^+^ Il1r1*^fl/fl^ mice (with proximal tubule–specific deletion of *Il1r1*) with or without LIPUS presented significantly milder kidney atrophy, tubular injury, and tubulointerstitial fibrosis compared with male *Pepck-Cre^–^ Il1r1*^fl/fl^ littermates (controls). Serum Cr levels and histological changes were similar between *Pepck-Cre^+^ Il1r1*^fl/fl^ mice with or without LIPUS (Figure 8, A–C). Additionally, expression levels of fibrotic markers (fibronectin and α-SMA), IL-1β downstream proteins (p-NF-κB and c-Myc), and tubular injury markers (*Havcr1* and *Lcn2*) were significantly lower in *Pepck-Cre^+^ Il1r1*^fl/fl^ mice with or without LIPUS than in the controls but comparable between the former 2 groups (Figure 8, D and E). Taken together, knockout of IL-1R in renal tubular proximal cells ameliorated renal fibrosis, and LIPUS might treat renal fibrosis by suppressing IL-1R activation.

## Discussion

We demonstrated that LIPUS attenuated IR-induced renal fibrosis in mice via inhibiting tubular partial EMT and the IL-1R signaling pathway. Interestingly, there were no significant differences in renal fibrosis and tubular injury between LIPUS-treated and untreated renal proximal tubule–specific *Il1r1*-knockout mice with renal IR injury.

Recently, nonthermal effects of ultrasonography have been increasingly highlighted (7, 21). Our results consistently showed a minimal thermal effect of LIPUS. LIPUS transmits acoustic energy to the target tissue, exerting pleiotropic therapeutic effects, such as regulating the immune response in experimental autoimmune myocarditis (22) and ameliorating angiotensin II–induced cardiac fibrosis (23). LIPUS has also been applied to treat AKI (10), hypertensive and diabetic nephropathy (8), and CKD (11). However, previous studies have rarely systematically evaluated parameter configurations for LIPUS in CKD models. To address this, we conducted in vivo and in vitro screening to identify an intensity of 315 mW/cm^2^ balancing biological efficacy and safety. We finally chose an intensity of 315 mW/cm^2^ for subsequent experiments.

LIPUS suppressed the levels of inflammation factors in mouse renal tissue, which is consistent with previous findings (10). Gigliotti et al. (24) have revealed that preexposure to ultrasound stimulation prevents IR-induced AKI via activating the splenic antiinflammatory pathway. However, LIPUS decreases tubulointerstitial fibrosis in experimental diabetic and hypertensive nephropathy models with the performance of splenectomy (8), suggesting that other pathways are involved in the preventive effect of LIPUS on renal fibrosis. LIPUS ameliorates the synovial inflammation by inhibiting the production of mature IL-1β (25). The tubular partial EMT was suppressed by LIPUS under the same concentration of IL-1β stimulation, indicating the inhibitory effect of LIPUS on the IL-1β downstream genes. COX-2 and prostaglandin E_2_ are downregulated by LIPUS in IL-1β–stimulated synovial cells, but the mechanism is still unknown (26).

Our data showed that LIPUS significantly downregulated IL-1R and its downstream genes in IR-induced renal tissue. ILs are direct effectors on renal function. For instance, inhibition of IL-1β benefits the treatment of renal tubular injury and fibrosis (15, 20, 27). A Mendelian randomization analysis uncovered a protective effect of IL-1R antagonists on renal function (28). Activated IL-1R in tubular epithelial cells exacerbates toxin-induced AKI and cell death (19, 29). Here, we observed tubular IL-1R involved in renal fibrosis was downregulated by LIPUS and no differences in tubular damage and renal fibrosis in IR-induced renal proximal tubule–specific *Il1r1*-knockout mice with or without LIPUS. Although our results suggest that LIPUS acts by suppressing IL-1R signaling, another possibility should be considered; that is, IL-1R knockout alone reduces injury so strongly that LIPUS provides no additional benefit. Future studies should include sham-treated IL-1R–knockout mice or test different injury levels in knockout mice to clarify LIPUS’s role.

Some studies suggest that prolonged isoflurane anesthesia may cause neurological damage, particularly in female, young, and aged rodents (30–32). However, we did not observe such effects in our study, possibly because each anesthesia session was relatively short and only adult male mice were used. Although all groups received the same dose of anesthesia, potential interactions between repeated isoflurane exposure and LIPUS should be further investigated in future studies. Moreover, nonfocused planar wave transducers were used in this study. No adverse effects were observed in adjacent organs; however, the precise energy absorption by these tissues remains unquantified and requires further investigation.

In conclusion, we examined the therapeutic efficacy of LIPUS on CKD by inhibiting the IL-1β/IL-1R pathway. We also screened a preferred parameter of LIPUS to treat CKD, providing references for its clinical application. Clinical interventional trials are needed in the future to confirm the efficacy of LIPUS in slowing the progression of CKD.

## Methods

### Sex as a biological variable.

This study employed male mice exclusively owing to established sex-specific differences in disease progression, where female mice demonstrate enhanced resistance to IR injury yet greater susceptibility to isoflurane-induced damage (32, 33). Consequently, the extrapolation of these findings to female individuals requires further validation.

### Animals.

Male C57BL/6 mice aged 8 weeks used in the present study were provided by the Animal Core facility of Nanjing Medical University. *Il1r1*-floxed mice (The Jackson Laboratory, stock no. 028398) were generated as described (34). We generated mice with specific deletion of IL-1R1 (*Pepck-Cre^+^ Il1r1*^fl/fl^) from epithelial cells in the proximal tubule by using the C57BL/6 *Pepck-Cre* mouse lines (35). Male *Pepck-Cre^–^ Il1r1*^fl/fl^ littermates (WT) were used as controls. Animals were housed in standard cages with a 12-hour light/12-hour dark cycle and given ad libitum feeding and drinking. Mice were randomly assigned to the control or experimental groups.

### LIPUS.

LIPUS was performed for 20 minutes using a nonfocused planar wave transducer (1 MHz frequency, spatial peak temporal average intensities: 25–600 mW/cm²). The ultrasound was delivered in 10 ms pulse bursts with 2 distinct duty cycle configurations: (a) 1% duty cycle (0.1 ms active phase of continuous 1 MHz waves followed by 9.9 ms interpulse interval) and (b) 10% duty cycle (1 ms active phase followed by 9 ms interval). Both configurations maintained a consistent pulse repetition frequency of 100 Hz (10 ms period) (Supplemental Figure 6).

For animal and cell experiments, the LIPUS transducers (models: KYI-F-16-01000 and KYI-F-34-01000, respectively; Nanjing KHONS Medtech Co., Ltd.) have effective radiating areas of 1 cm² and 5 cm², measured according to International Electrotechnical Commission 61689:2013 standard, with modifications. In vivo, the LIPUS transducers were positioned on the dorsal skin surface overlying both kidneys (left and right), with an approximate distance of 3 mm between the probe and renal parenchyma (accounting for skin and subcutaneous tissue thickness). Each kidney received identical treatment parameters. For treatment planning, depth is approximated based on the tissue’s acoustic attenuation coefficient and transducer frequency. Using the characteristic attenuation coefficient for skin and soft tissue (1 dB/cm/MHz), the depth at which acoustic intensity attenuates to 50% of its initial value is calculated as 3 cm.

### Experimental protocols.

A CKD mouse model was created by unilateral IR-induced renal fibrosis. In brief, without the performance of contralateral nephrectomy, the left renal pedicle was clamped to allow a 35-minute ischemia, followed by the release of the microvascular clamp to allow reperfusion. During the IR surgery, the body temperature of mice was controlled at 36.5°C–37.5°C (13). Intraoperative injection of recombinant IL-1β (rIL-1β, catalog 401-ML, R&D Systems, Bio-Techne) 10 ng/g was conducted where indicated. All mice were euthanized and sacrificed on day 14 after renal IR and/or IL-1β injection.

On the day after IR surgery or rIL-1β injection, mice were anesthetized with isoflurane for LIPUS (Nanjing KHONS Medtech Co., Ltd.). Briefly, mice were shaved and placed in the prone position, and the prewarmed gel was then applied to the depilated skin on the back for LIPUS (Supplemental Figure 6).

LIPUS was performed with an intensity of 315 mW/cm^2^ and 1% duty per day or every other day from day 1 to 14. Serum and kidney samples were harvested, and serum Cr in mice was measured using an automatic biochemical analyzer (Chemray-240, Rayto Inc.). Supplemental Figure 3B demonstrates serum Cr measured 24 hours following surgical removal of the contralateral kidney. To evaluate the systemic and local safety of LIPUS, peripheral blood was collected for complete blood count (hemoglobin, white blood cells, platelets) and liver function analysis (ALT, AST) using an automated hematology analyzer (Mindray, BC-2800vet, ServiceBio) and enzymatic assays (Chemray-240), respectively. Additionally, skin tissue (probe contact site) and liver tissue were harvested, fixed in 4% paraformaldehyde, and H&E-stained to histologically assess local toxicity or inflammation.

All experimental groups, including controls, received identical isoflurane exposure to maintain consistency. Control mice underwent sham surgery with the same anesthesia protocol as treatment groups.

### LIPUS treating MTECs and podocytes in vitro.

Cells cultured in standard polystyrene plates (catalog 706001, NEST) received LIPUS treatment through the plate supporting the culture dishes (Supplemental Figure 6), with acoustic transmission analysis confirming signal attenuation (1.46 dB, corresponding to 29% intensity reduction).

Following serum starvation, MTECs (catalog CCL-139, ATCC) were exposed to recombinant human TGF-β1 (catalog AF-100-21C, Peprotech) at 15 ng/mL or rIL-1β at 5, 10, and 20 pg/μL. IL-1β at 10 pg/μL was chosen as an optimal concentration to induce partial tubular EMT. Subsequently, the culture plate was placed on sterile water and exposed to LIPUS at an intensity of 25, 102, 315, and 410 mW/cm^2^ and 1% or 10% duty cycle for 20 minutes. The temperature of the culture media in the plate was monitored using a temperature indicator stripe (TMC Hallcrest). Twenty-four hours after 20-minute LIPUS pretreatment and 10 pg/μL IL-1β exposure, cells were harvested for Western blotting and real-time quantitative PCR (RT-qPCR). To detect the protein level of p-NF-κB, MTECs underwent the same procedures but were exposed to IL-1β for 15 minutes.

The human podocyte cell line was provided by M. Saleem (Children’s Renal Unit, Bristol Royal Hospital for Children, University of Bristol, Bristol, United Kingdom). Podocytes were cultured as described previously (36). Briefly, cells were proliferated at 33°C and differentiated at 37°C. Differentiated podocytes were exposed to LIPUS at intensities of 25, 102, 315, and 600 mW/cm² with a 1% duty cycle for 20 minutes each.

### Flow cytometry.

Viable cells and cell debris were collected and incubated with propidium iodide and annexin V fluorescein isothiocyanate (catalog 556547, BD Biosciences) in the dark, followed by semiquantification of apoptotic cells using flow cytometry. The percentage of apoptotic cells in late (upper-right quadrant in Q2) and early apoptosis (lower-right quadrant in Q3) was calculated.

### Histological staining.

All collected tissues (kidney, liver, and probe-contact skin) underwent 24-hour fixation in 4% paraformaldehyde at 4°C, followed by standard paraffin-embedding and sectioning at 3 μm thickness. Renal tissues were subjected to PAS and Masson’s trichrome staining, while hepatic and skin tissues were processed for H&E staining. Observation was under a Leica DM3000 microscope. Semiquantitative measurements of the tubular injury and fibrosis scores on an ordinal scale were performed in a blinded manner.

### RT-qPCR.

Total RNA was extracted from renal tissue or cells using FastPure Cell/Tissue Total RNA isolation kit (catalog RC112, Vazyme) and quantified by a NanoDrop Spectrophotometer (NanoDrop Technologies). After reverse transcription using HiScript III All-in-one RT SuperMix Perfect for qPCR (catalog R333, Vazyme), RT-qPCR was performed using ChamQ SYBR qPCR Master Mix (catalog Q321, Vazyme) in triplicate. Quantification of the target gene was normalized to that of glyceraldehyde-3-phosphate dehydrogenase (*Gapdh*), then calculated using the 2^−ΔΔCt^ method. Sequences of primers used in RT-qPCR are listed in Supplemental Table 1.

### Western blotting.

Protein sample preparation and Western blotting were carried out as previously described (37). Primary antibodies of anti-fibronectin (catalog F3648, Sigma-Aldrich), anti-α-SMA (catalog ab7817, Abcam), anti-IL-1R (catalog sc-393998, Santa Cruz Biotechnology), anti-p-NF-κB p65 (Ser536) (catalog 3033, Cell Signaling Technology), anti-Myd88 (catalog sc-74532, Santa Cruz Biotechnology), and anti-c-Myc (catalog 10828, Proteintech), as well as secondary antibodies of HRP-conjugated goat anti-mouse IgG (catalog ab6789, Abcam) and HRP-conjugated goat anti-rabbit IgG (catalog ab6721, Abcam), were used.

### Immunofluorescence.

Immunofluorescence staining was performed and quantified on frozen renal sections as described previously (13). Briefly, frozen renal sections in 3 μm thickness were blocked for nonspecific antigens and incubated overnight at 4°C with the primary antibodies of anti-α-SMA (catalog ab7817, Abcam). After incubation with secondary antibodies for 1 hour at room temperature, one drop of DAPI-Fluoromount-G (catalog 0100-20, SouthernBiotech) was applied to the frozen section to counterstain the nuclear DNA. Ten random fields per section were observed under a fluorescence microscope (DM3000, Leica Microsystems) (original magnification 400×) for quantitative measurement of positive staining of α-SMA using ImageJ (NIH).

### Immunohistochemistry.

Paraffin-embedded tissue sections (3 μm) were deparaffinized with xylene and rehydrated through a graded ethanol series, followed by antigen retrieval in heated citrate buffer (pH 6.0, 95°C) for 20 minutes. After blocking nonspecific binding with 5% bovine serum albumin (room temperature, 1 hour), sections were incubated overnight at 4°C with primary antibodies against F4/80 (catalog GB113373, ServiceBio) and Ly6G (catalog GB11229, ServiceBio). Following secondary HRP antibody incubation (catalog ab6789, or catalog ab6721, Abcam) (37°C, 1 hour), DAB development (2 minutes) (MXB Biotechnologies, DAB-0031), hematoxylin counterstaining, and dehydration, sections were mounted. Ten randomly selected fields at 200× original magnification (Axioscan 7 system, ZEISS) were analyzed per tissue section using ImageJ, and the F4/80-positive areas or Ly6G-positive cells were quantified.

### ELISA.

Tissue homogenates were collected for quantifying IL-1β levels using the commercial ELISA kit (catalog EMC001b, Neobioscience Technology) according to the manufacturer’s instructions.

### RNA sequencing.

Total RNA from mouse renal tissue was extracted using TRIzol reagent (Invitrogen, Thermo Fisher Scientific), qualified by the 5300 Bioanalyzer (Agilent), and quantified by the NanoDrop2000 spectrophotometer (NanoDrop Technologies). RNA purification, reverse transcription, library construction, and sequencing were performed at Shanghai Majorbio Bio-pharm Biotechnology Co., Ltd, according to the manufacturer’s instructions (Illumina). To identify DEGs with|log_2_FC|≥ 0.58 and FDR< 0.05 (DESeq2) between the 2 samples, the expression level of each transcript was determined according to the transcripts per million reads. In addition, significantly enriched pathways in DEGs were screened by the gene set enrichment analysis of KEGG at a Benjamini-Hochberg–adjusted *P* < 0.05 versus the whole-transcriptome background. All data were analyzed on the Majorbio Cloud Platform (https://cloud.majorbio.com/).

### Statistics.

Data were collected in a blinded manner. The number of animals was chosen to ensure adequate statistical power and was based on previous experience with CKD animals and knockout mice. Statistical analyses were conducted using GraphPad Prism (version 9, GraphPad Software). Quantifications of images were performed by ImageJ or Image-Pro Plus (Media Cybernetics). Data are expressed as mean ± SD. Student’s 2-tailed *t* test compared 2 groups. One-way ANOVA with Tukey’s post hoc test analyzed multigroup comparisons. For factorial designs, 2-way ANOVA with Šídák’s multiple comparisons test was performed. In all tests, a 2-tailed α-level value of less than 0.05 was considered statistically significant. *P* < 0.05 was considered statistically significant. All statistical details regarding *P* value and *n* are indicated in the main and supplemental figure legends.

### Study approval.

All animal procedures were approved by Nanjing Medical University Institutional Animal Care and Use Committee (IACUC-2404046).

### Data availability.

RNA-Seq data used in this study are deposited in NCBI SRA BioProject (accession ID PRJNA1282700; https://www.ncbi.nlm.nih.gov/sra/PRJNA1282700). The datasets generated and analyzed during this study are included in the Supporting Data Values file.

## Author contributions

ZH and JD designed research studies, conducted experiments, acquired data, and analyzed data. ZH wrote the manuscript. ZF conducted experiments, acquired data, and analyzed data. LL, SL, LW, HG, AB, and KL conducted experiments. WS, SDC, JR, and XK provided reagents. SDC, JR, CX, XK, and HM reviewed and edited the manuscript. HM designed research studies. JR, XK, and HM provided experimental supervision. ZH (first), JD (second), and ZF (third) are co–first authors based on their degrees of contribution.

## Supplementary Material

Supplemental data

Unedited blot and gel images

Supporting data values

## Version 1 07/29/2025

In-Press Preview

## Version 2 09/09/2025

Electronic publication
