# Supplementary material for: Low-intensity pulsed ultrasound stimulation to treat renal fibrosis through inhibiting tubular IL-1R
Source: JCI Insight. 2025 Jul 29;10(17):e186892. doi: 10.1172/jci.insight.186892 (PMC12487682; doi:10.1172/jci.insight.186892)
Supplement: Supplemental data [file jciinsight-10-186892-s021.pdf]

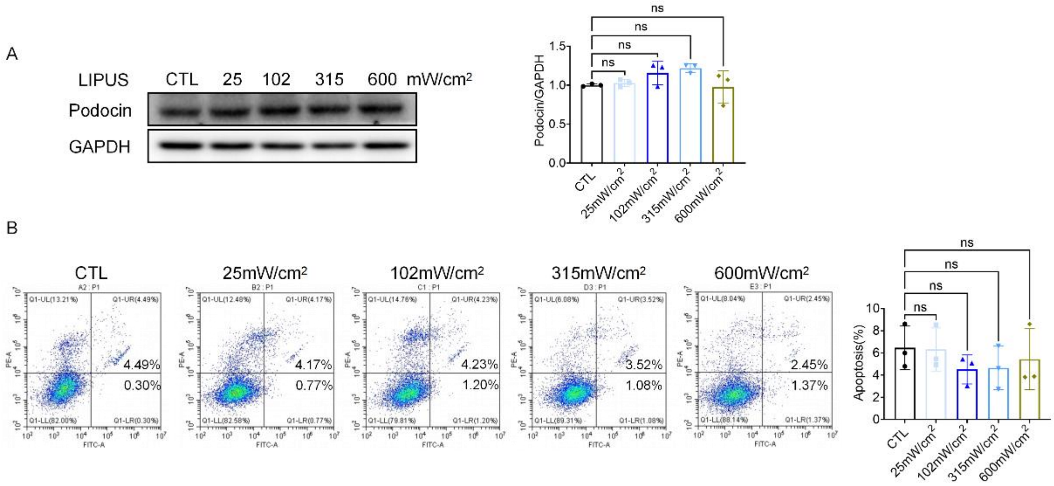

Supplemental Figure 1 The impact of LIPUS on podocytes.
(A) Protein level of podocin in podocytes, and the quantitative analysis (*n*=3). **(B)** Apoptosis of podocytes and quantification of apoptotic cells (Q2 + Q3) (*n*=3). Data information: Data are presented as mean±SD. Data were analyzed by one-way ANOVA with Tukey's post-hoc test. ns, no significant difference.

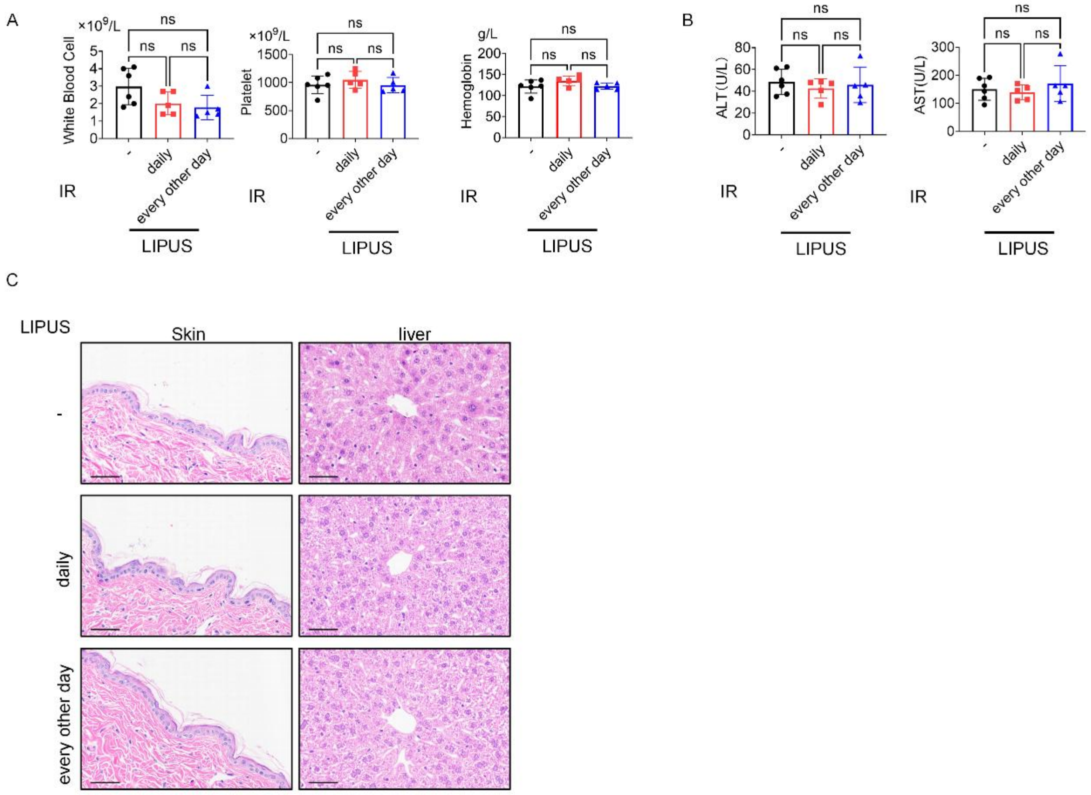

Supplemental Figure 2 Assessment of LIPUS safety in vivo.

(A) Changes in leukocyte counts, platelet counts, and hemoglobin concentrations across groups. **(B)** Effects of LIPUS on liver function markers. **(C)** Representative H&E staining images of transducer-contact skin and hepatic parenchyma. Scale bar: 50 μm. Data information: Data are presented as mean±SD. Statistical analysis was performed using one-way ANOVA with Tukey's post-hoc test. ns, no significant difference. *n*=6 in IR-induced renal injury group, *n*=5 in IR-induced renal injury group with every other day LIPUS treatment, *n*=5 in IR-induced renal injury group with daily LIPUS treatment.

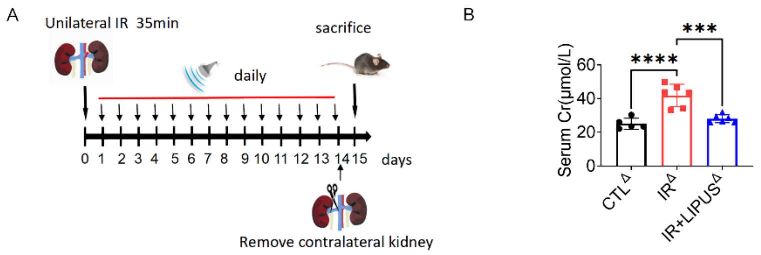

Supplemental Figure 3 Protective role of LIPUS in an IR-induced CKD model with contralateral nephrectomy.

(A) Study protocol. **(B)** Serum Cr levels in IR-induced mice with LIPUS after contralateral nephrectomy. Data information: Data are presented as mean±SD. One-way ANOVA with Tukey's post-hoc test was used for statistics. ***P<0.001, ****P<0.0001. n=5 in control$^{\Delta}$ group with contralateral nephrectomy, n=6 in IR$^{\Delta}$ group and IR treated with LIPUS group (IR+LIPUS$^{\Delta}$) with contralateral nephrectomy.

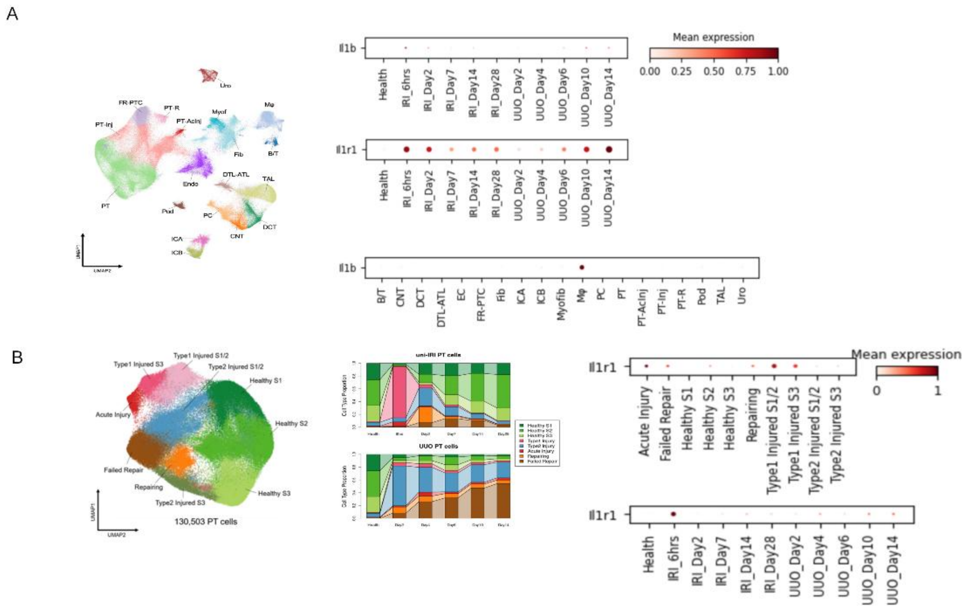

Supplemental Figure 4 Kidney interactive transcriptomics of genes involved in CKD.

(A) Integrated single-cell transcriptome map of mouse kidneys with blank control, unilateral IR injury, and unilateral ureteral obstruction. Dot plot of single-cell RNA sequencing analyses showing the expression levels of *Il1b* and *Il1r1* at various time points in mouse kidneys, and *Il1b* in multiple cell lineages. **(B)** UMAP plots of all proximal tubule cells in sub-clustering analysis. Connected bar plots displaying the proportional abundance of each cell cluster in each disease condition. Dot plot of single-cell RNA sequencing analyses showing the up-regulation of *Il1r1* in injured proximal tubules at 6 h, 14 d, and 28 d after unilateral IR injury, and 14 d after unilateral ureteral obstruction.

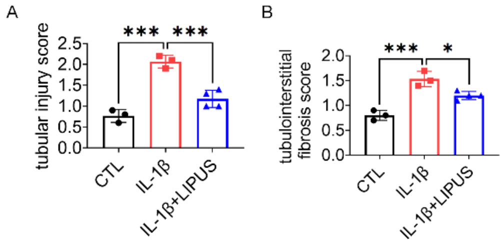

Supplemental Figure 5 Batch 2 replication: LIPUS-mediated protection against tubular injury and interstitial fibrosis in IL-1β-induced renal damage

(A) Tubular injury scores. (n=3-4) **(B)** Tubulointerstitial fibrosis scores. (n=3-4) Data information: Data are presented as mean±SD, with significance determined by one-way ANOVA with Tukey's post hoc test. *P<0.05, ***P<0.001.

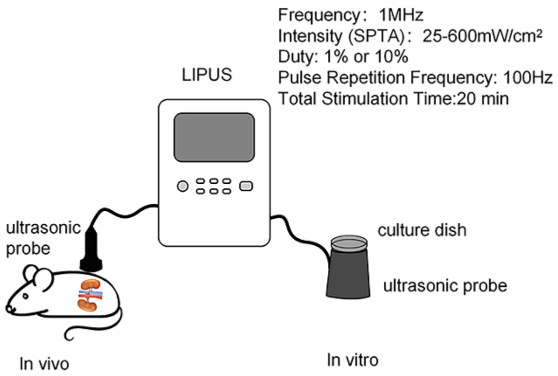

Supplemental Figure 6 Schematic of Low-Intensity Pulsed Ultrasound (LIPUS) stimulation. Simplified unilateral depiction of LIPUS application. In actual experiments, bilateral kidneys were treated using two transducers (one per kidney), each delivering 20 min of continuous exposure.

Supplemental Table 1 Sequences of primers used in RT-qPCR

gene name	primer sequences
Gapdh	F:CATCACTGCCACCCAGAAGACTG
	R:ATGCCAGTGAGCTTCCCGTTCAG
Fn1	F:CCGTGGGATGTTTGAGACTT
	R:GGCAAAAGAAAGCAGAGGTG
Acta2	F:CGGGAGAAAATGACCCAGATT
	R:AGGGACAGCACAGCCTGAATAG
Tgfb1	F:TGAGTGGCTGTCTTTTGACG
	R:TCTCTGTGGAGCTGAAGCAA
Serpine1	F:AGTCTTTCCGACCAAGAGCA
	R:ATCACTTGCCCCATGAAGAG
Lcn2	F:ATGTCACCTCCATCCTGGTCAG
	R:GCCACTTGCACATTGTAGCTCTG
Havcr1	F:ACATATCGTGGAATCACAACGAC
	RACTGCTCTTCTGATAGGTGACA
Il1r1	F:GTGCTACTGGGGCTCATTTGT
	R:GGAGTAAGAGGACACTTGCGAAT
Tnf	F:CAGGCGGTGCCTATGTCTC
	R:CGATCACCCCGAAGTTCAGTAG
Il6	F:TACCACTTCACAAGTCGGAGGC
	R:CTGCAAGTGCATCATCGTTGTTC
Ccl2	F:GCTACAAGAGGATCACCAGCAG
	R:GTCTGGACCCATTCCTTCTTGG
