# Supplementary material for: Low-intensity pulsed ultrasound stimulation to treat renal fibrosis through inhibiting tubular IL-1R
Source: JCI Insight. 2025 Jul 29;10(17):e186892. doi: 10.1172/jci.insight.186892 (PMC12487682; doi:10.1172/jci.insight.186892)

The blots used for the semiquantitative densitometric analysis

Figure 1D α-SMA

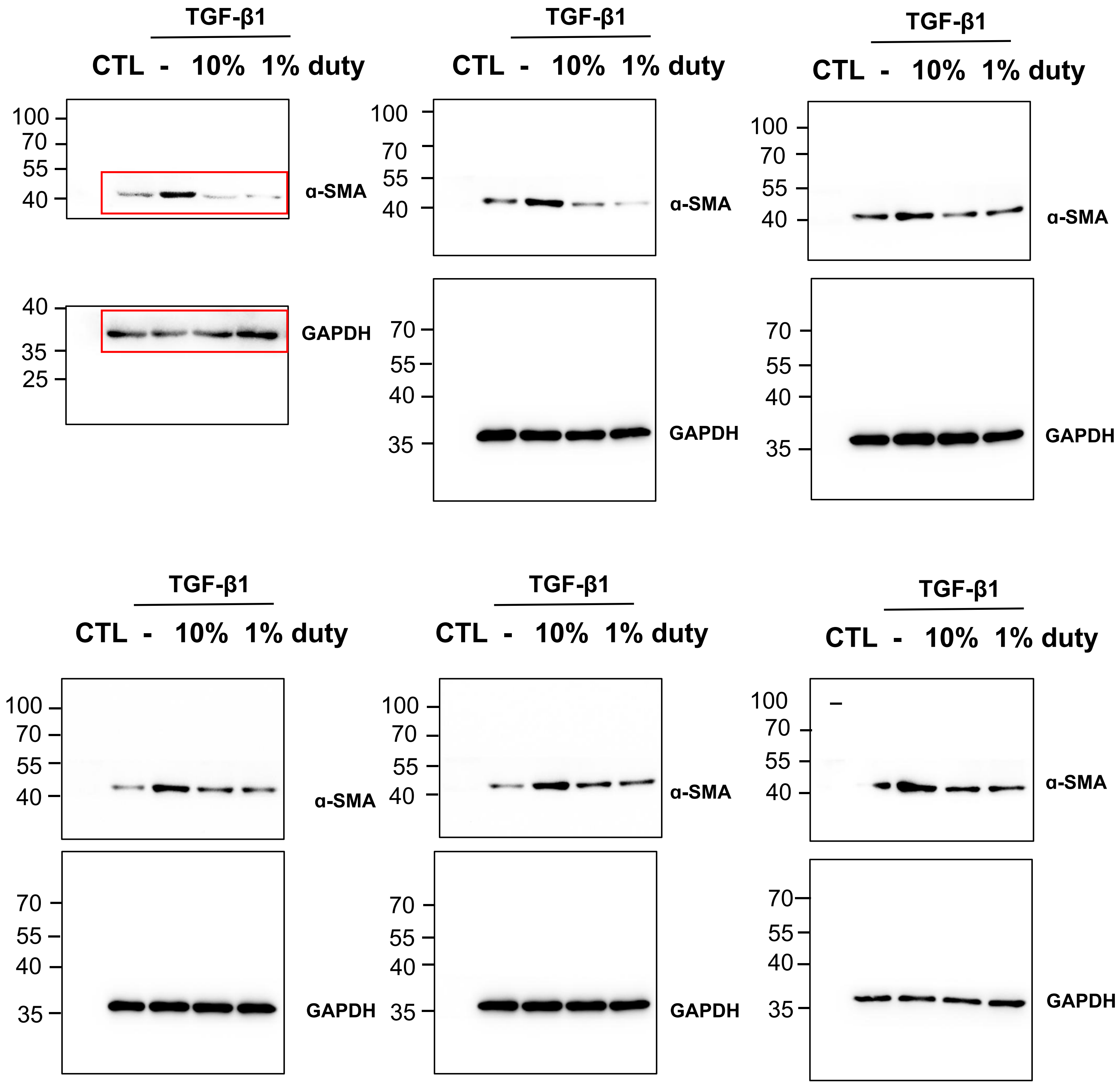

Figure 1E α-SMA

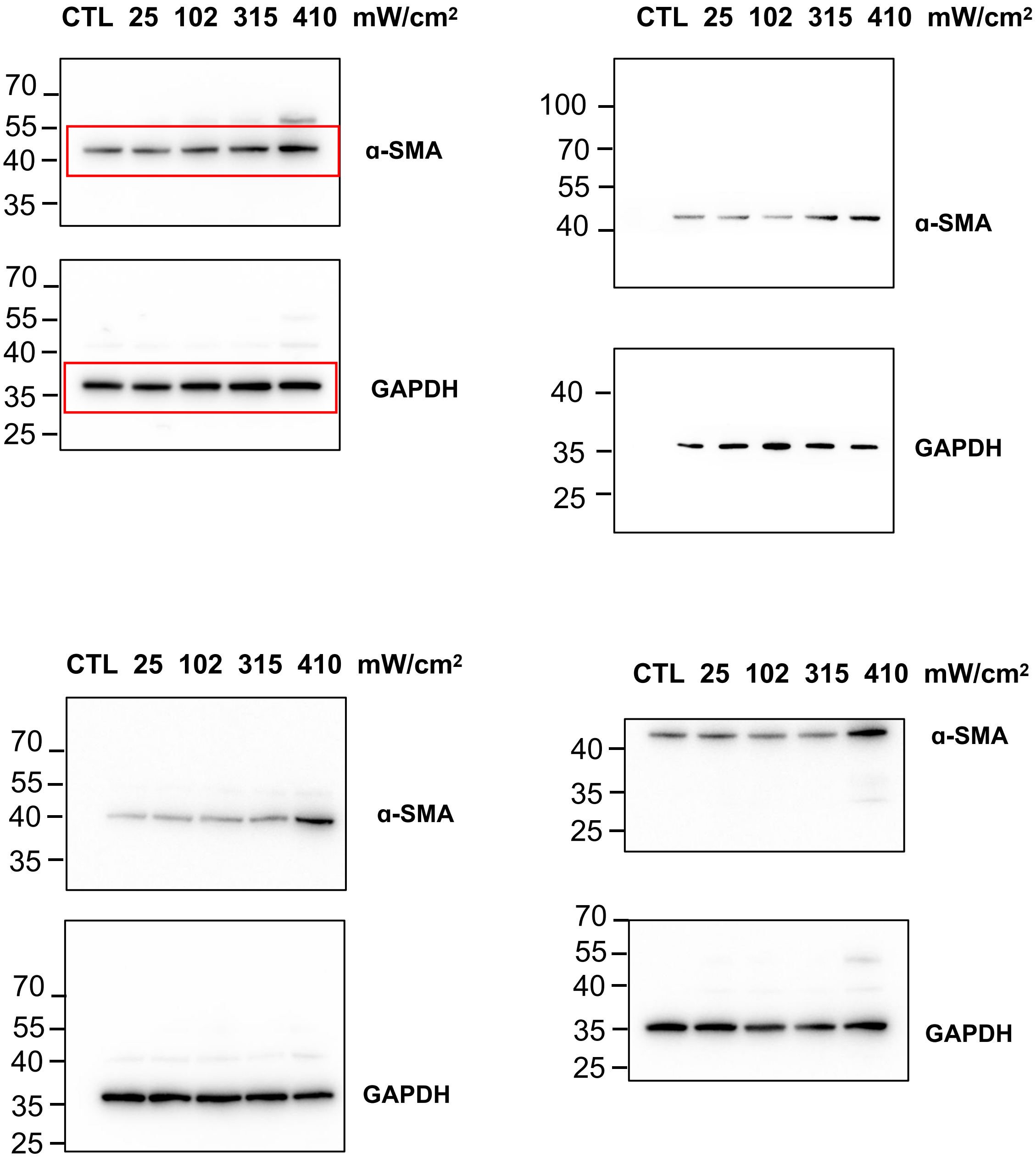

Figure 1F α-SMA

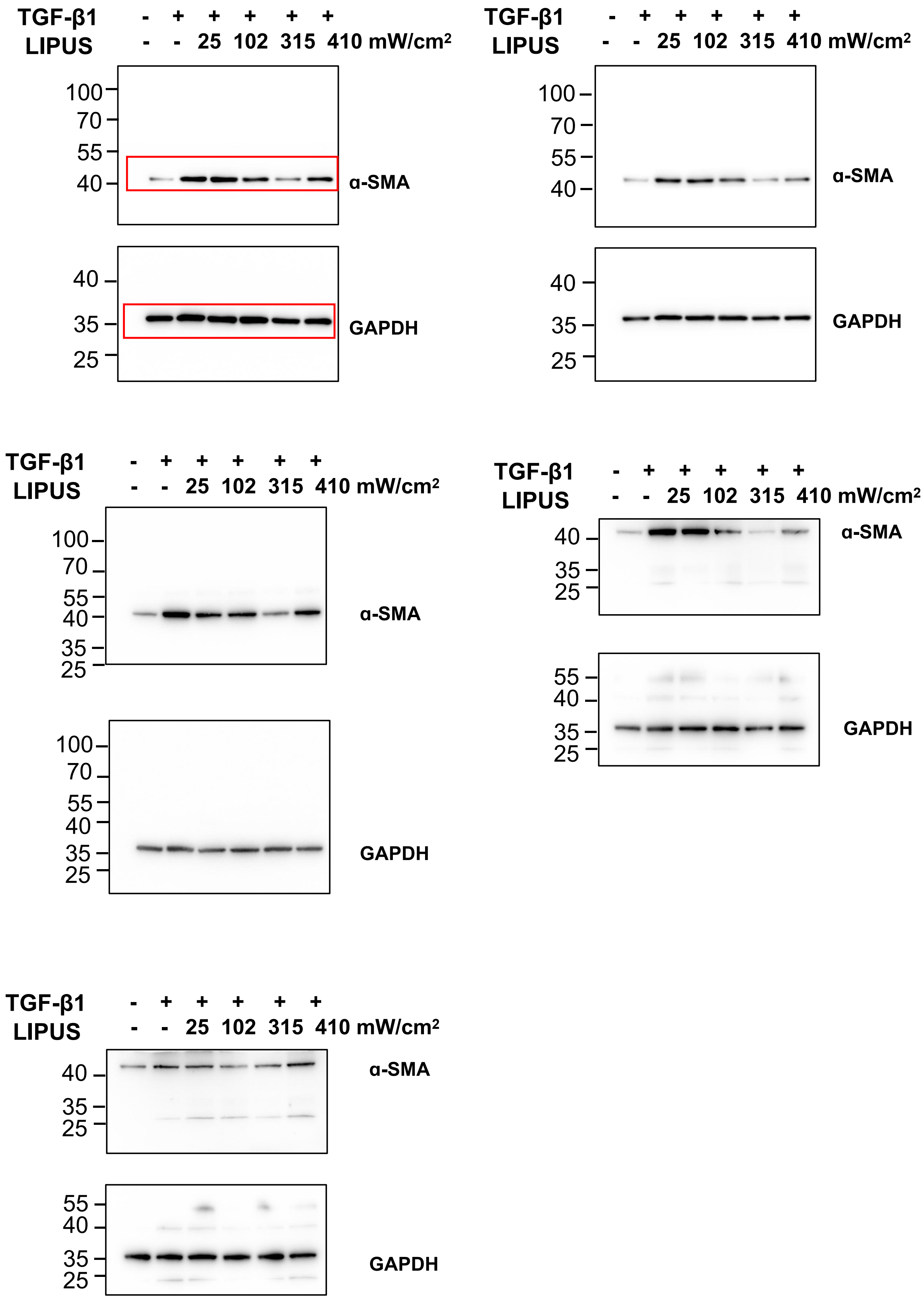

Figure 3A Fibronectin & α-SMA

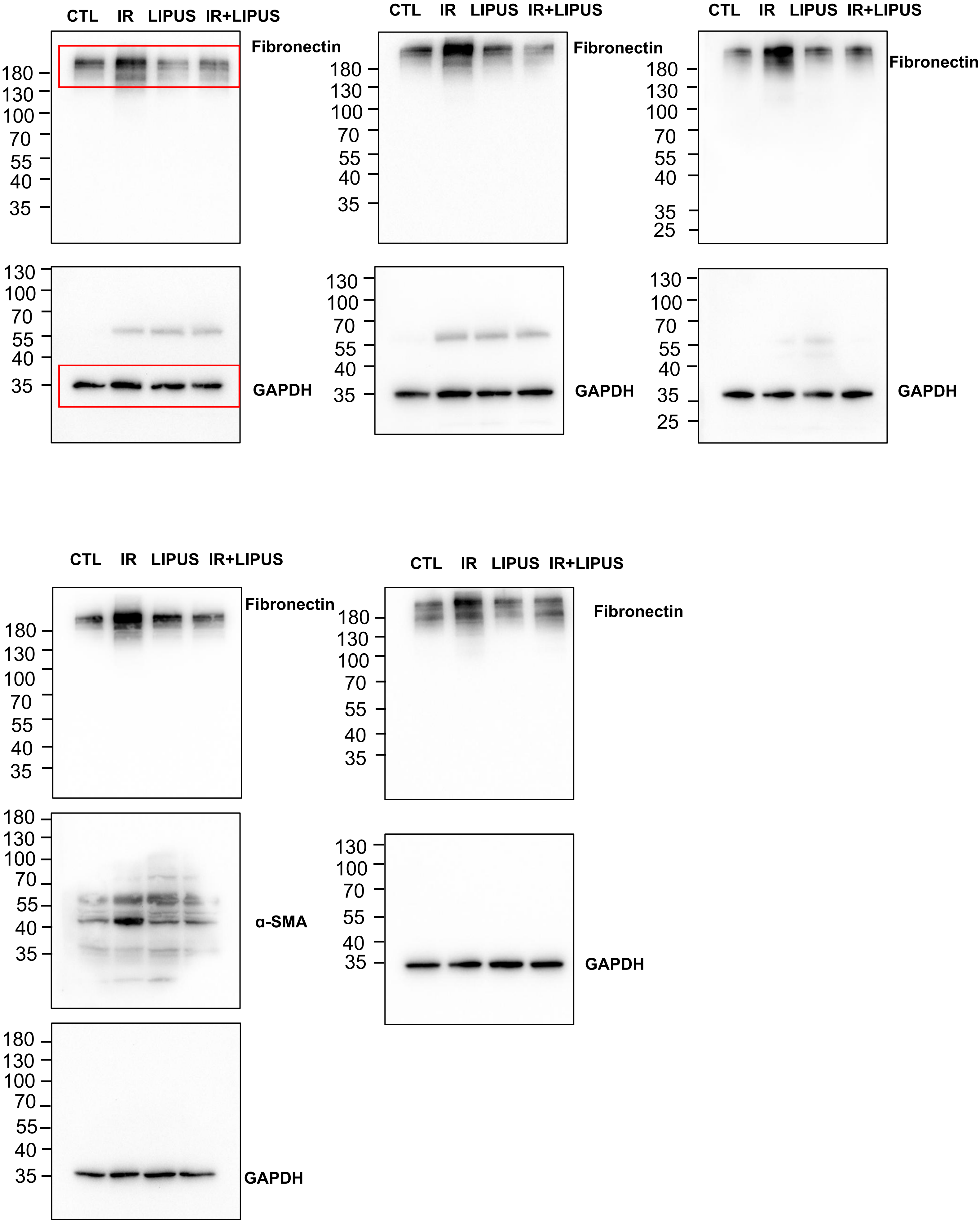

Figure 3A Fibronectin & α-SMA

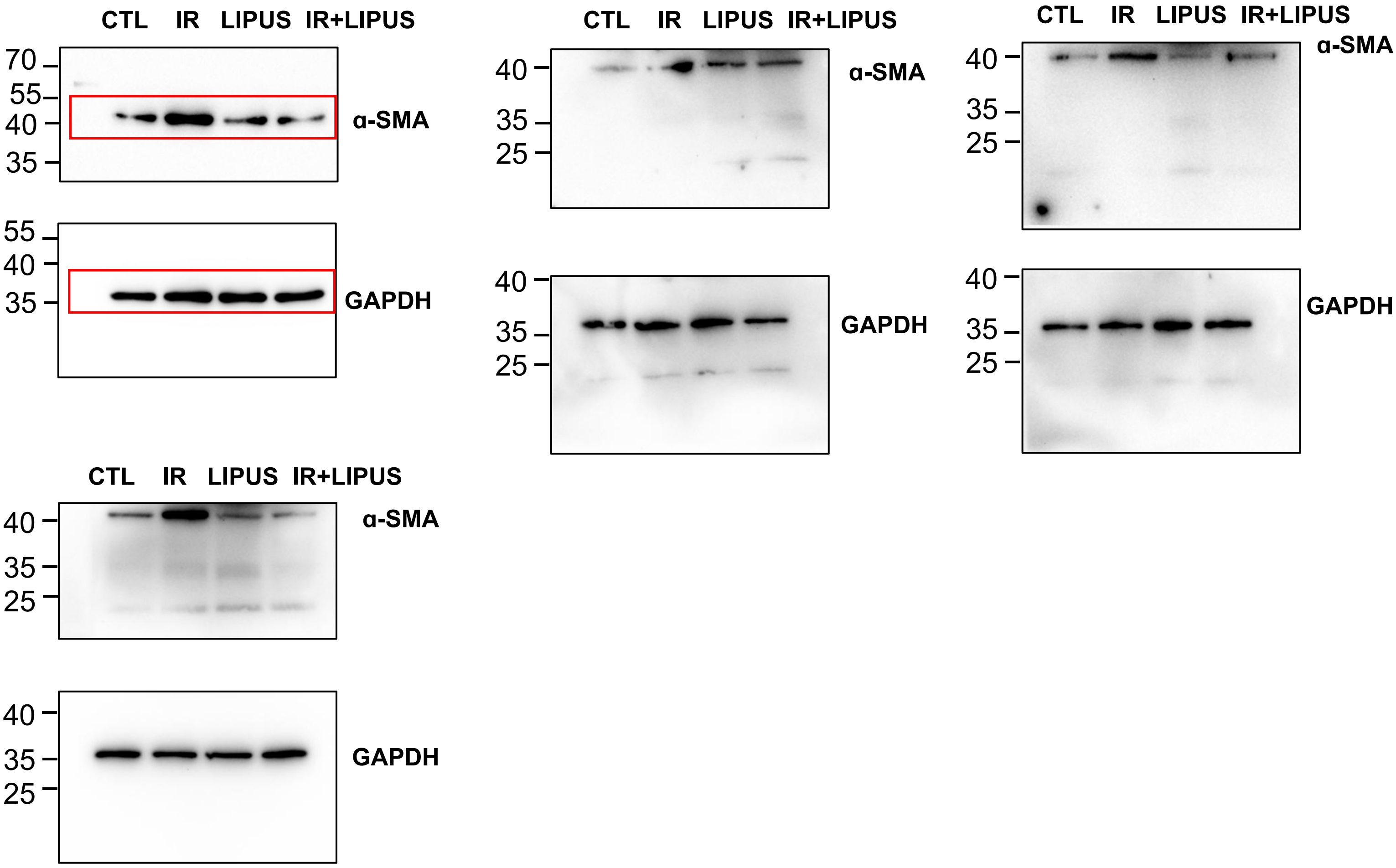

Figure 4A IL-1R

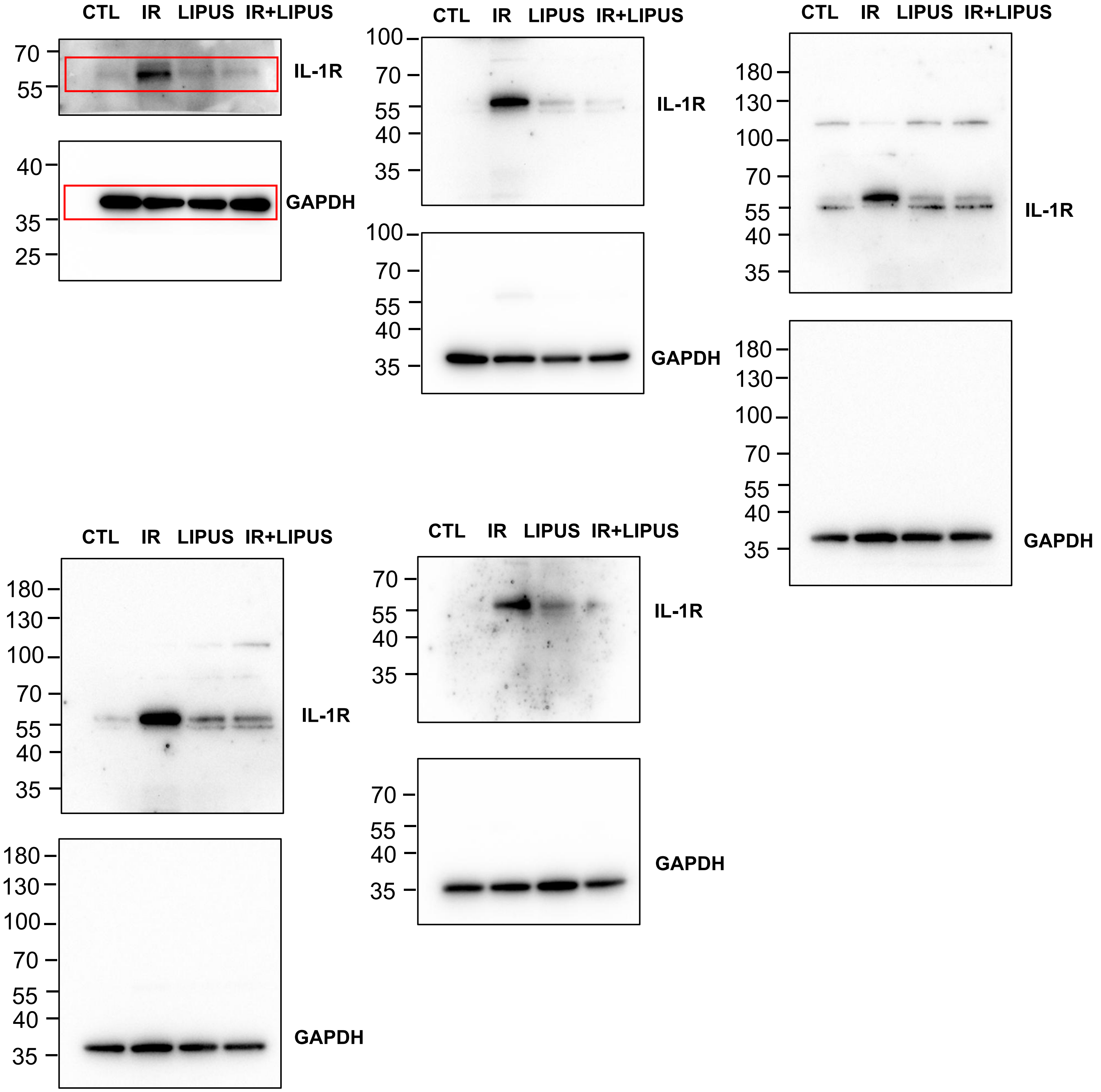

Figure 4B Myd88 & p-NF-κB & c-Myc

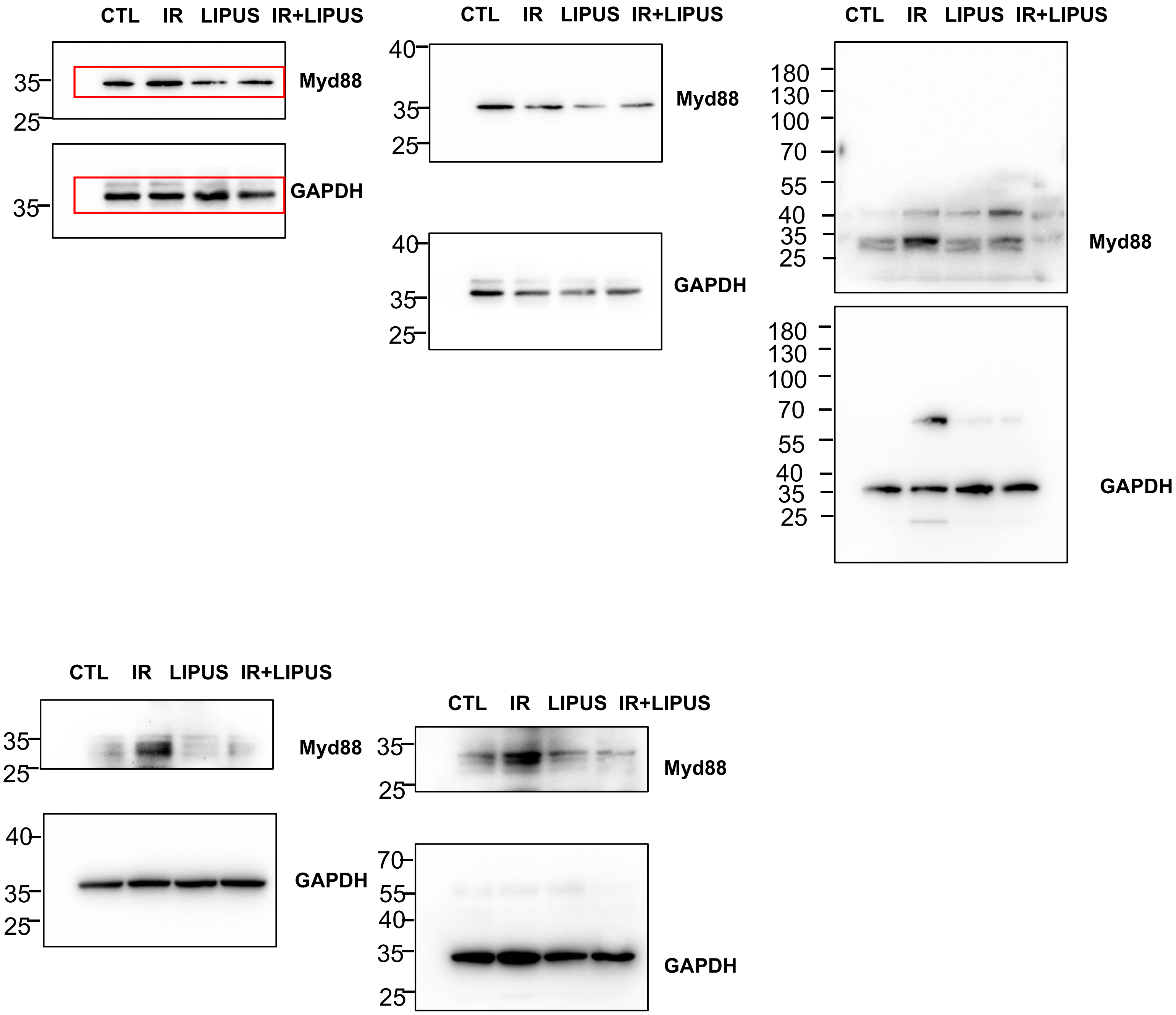

Figure 4B Myd88 & p-NF-κB & c-Myc

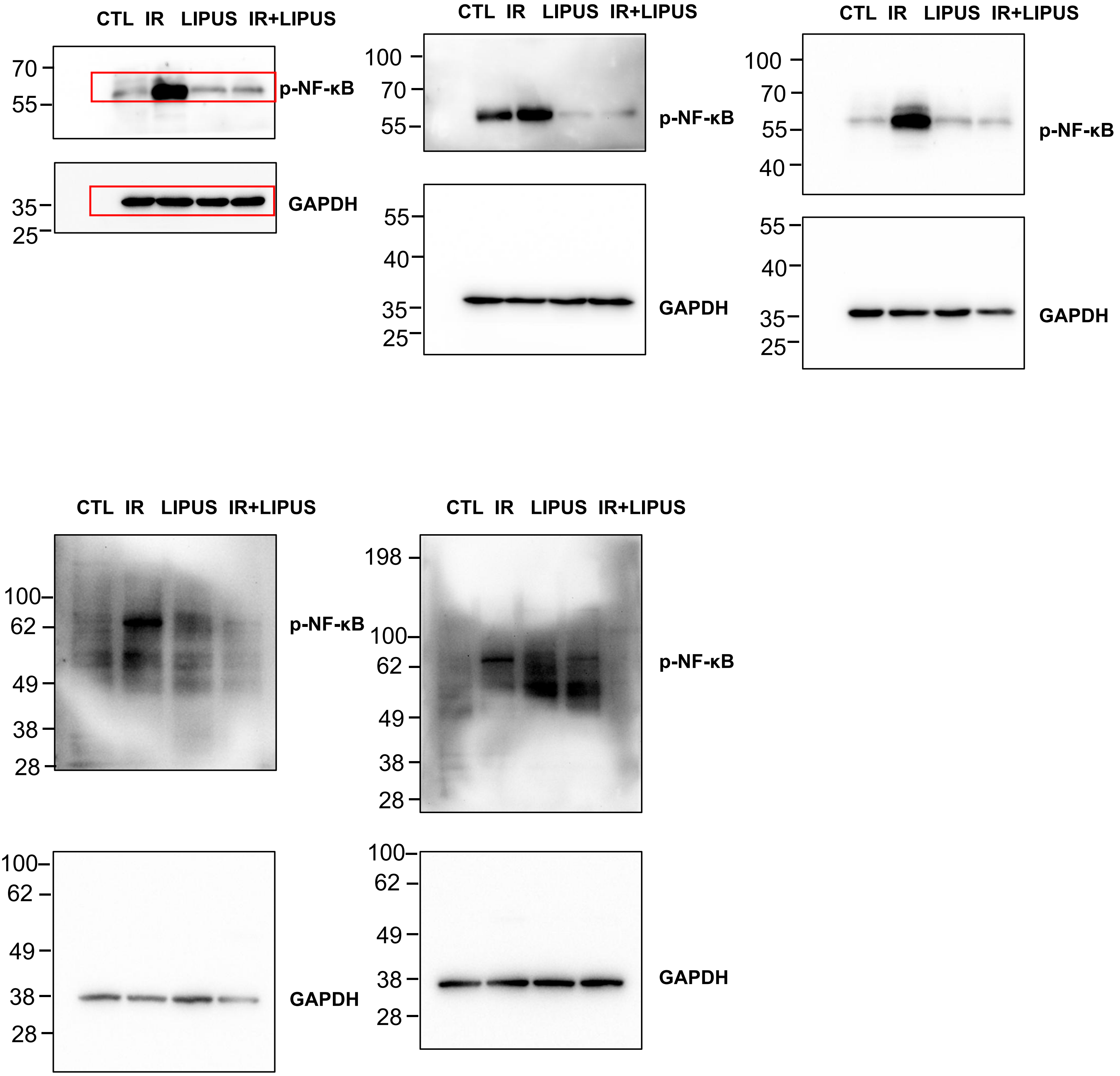

Figure 4B Myd88 & p-NF-κB & c-Myc

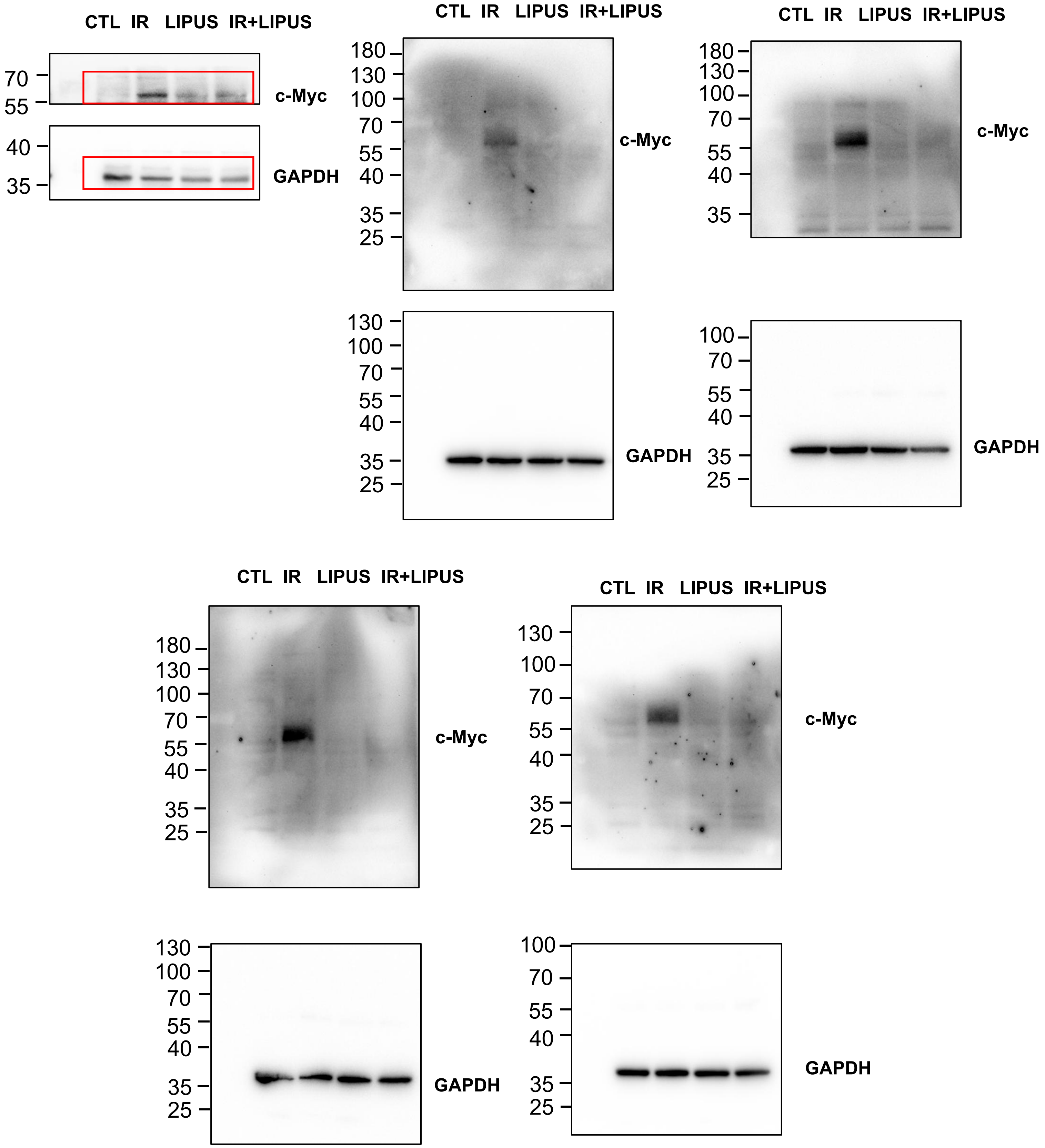

Figure 4C α-SMA

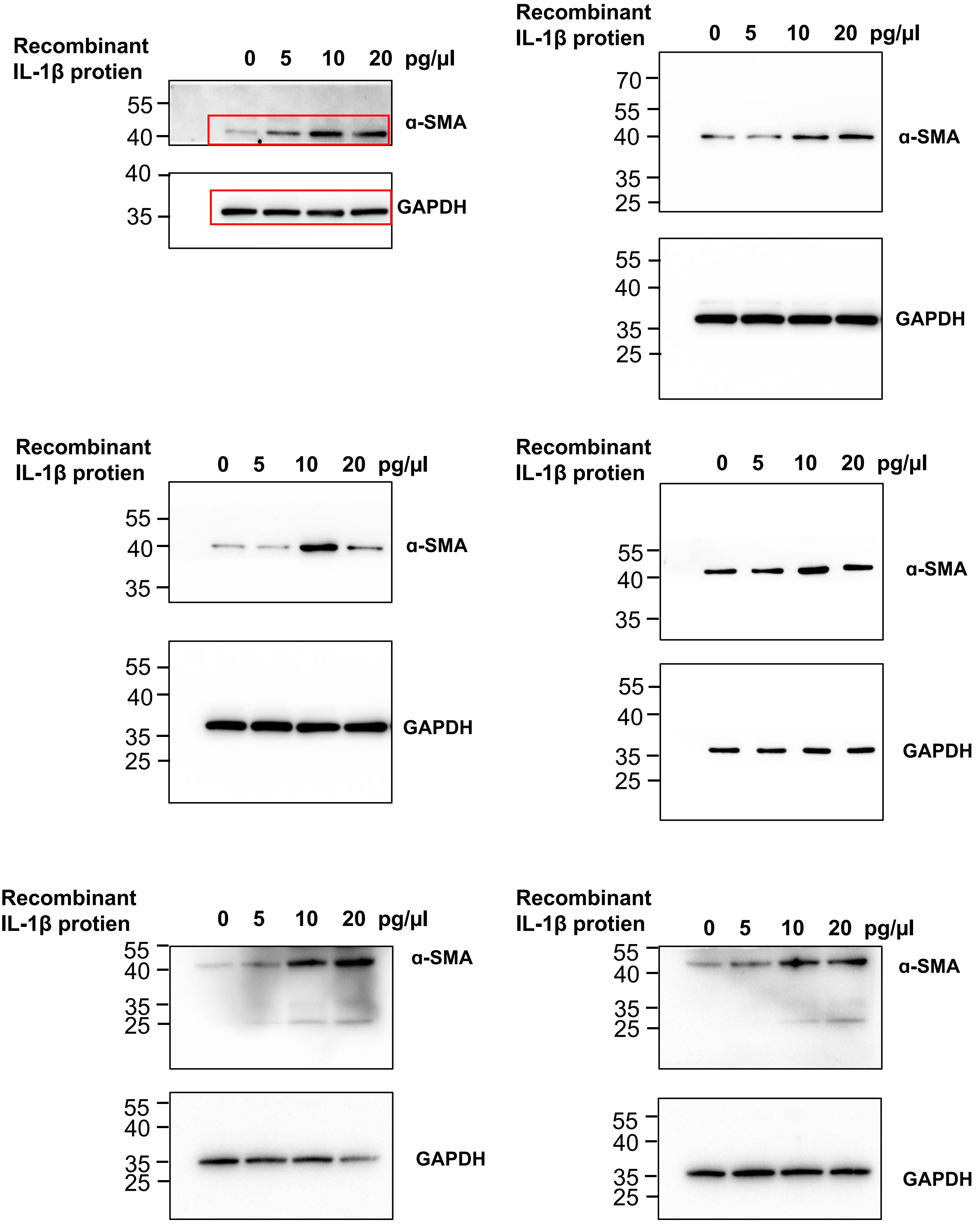

Figure 4D α-SMA & Fibronectin

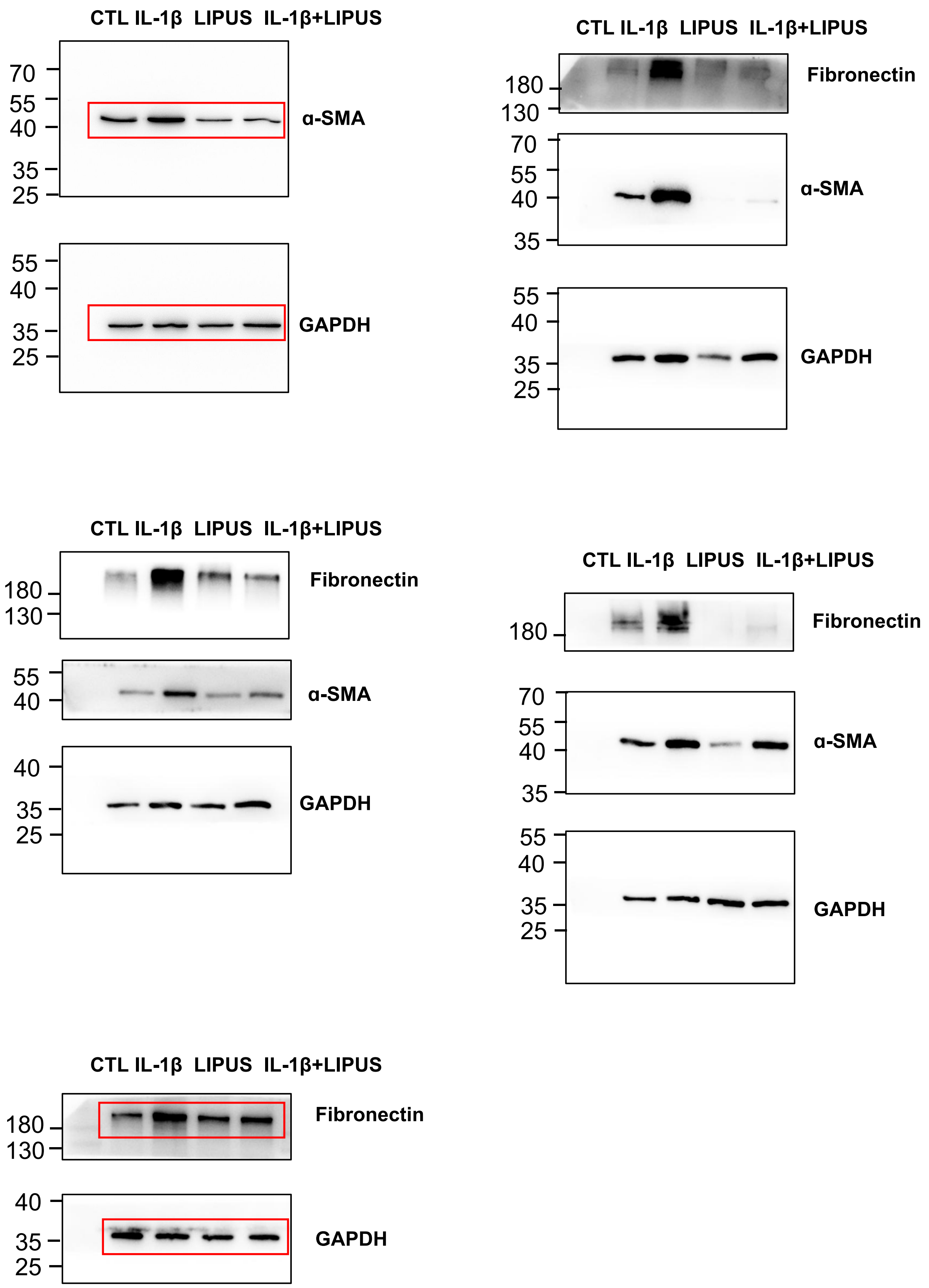

Figure 4E IL-1R & p-NF-κB & c-Myc

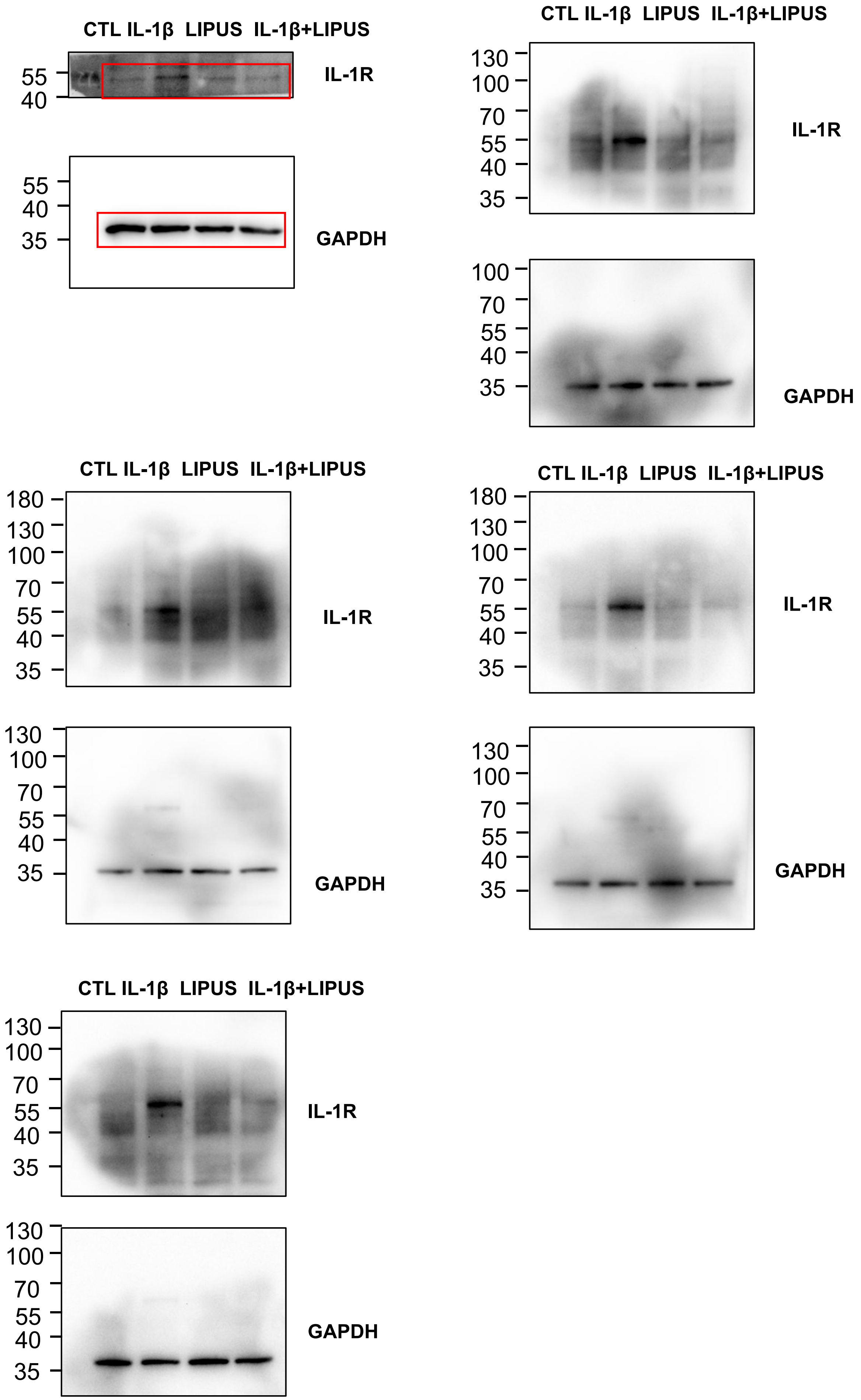

Figure 4E IL-1R & p-NF-κB & c-Myc

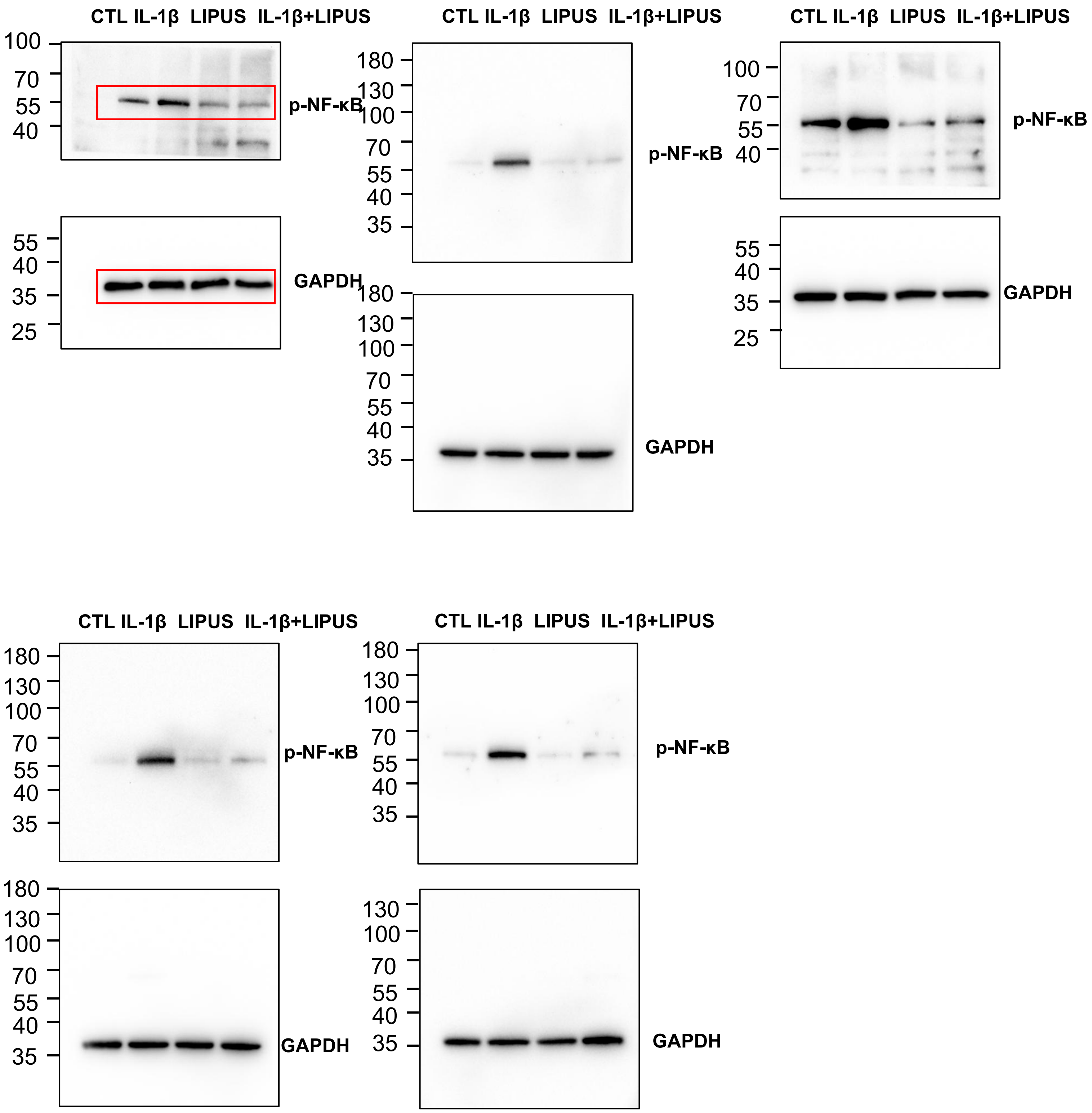

Figure 4E IL-1R & p-NF-κB & c-Myc

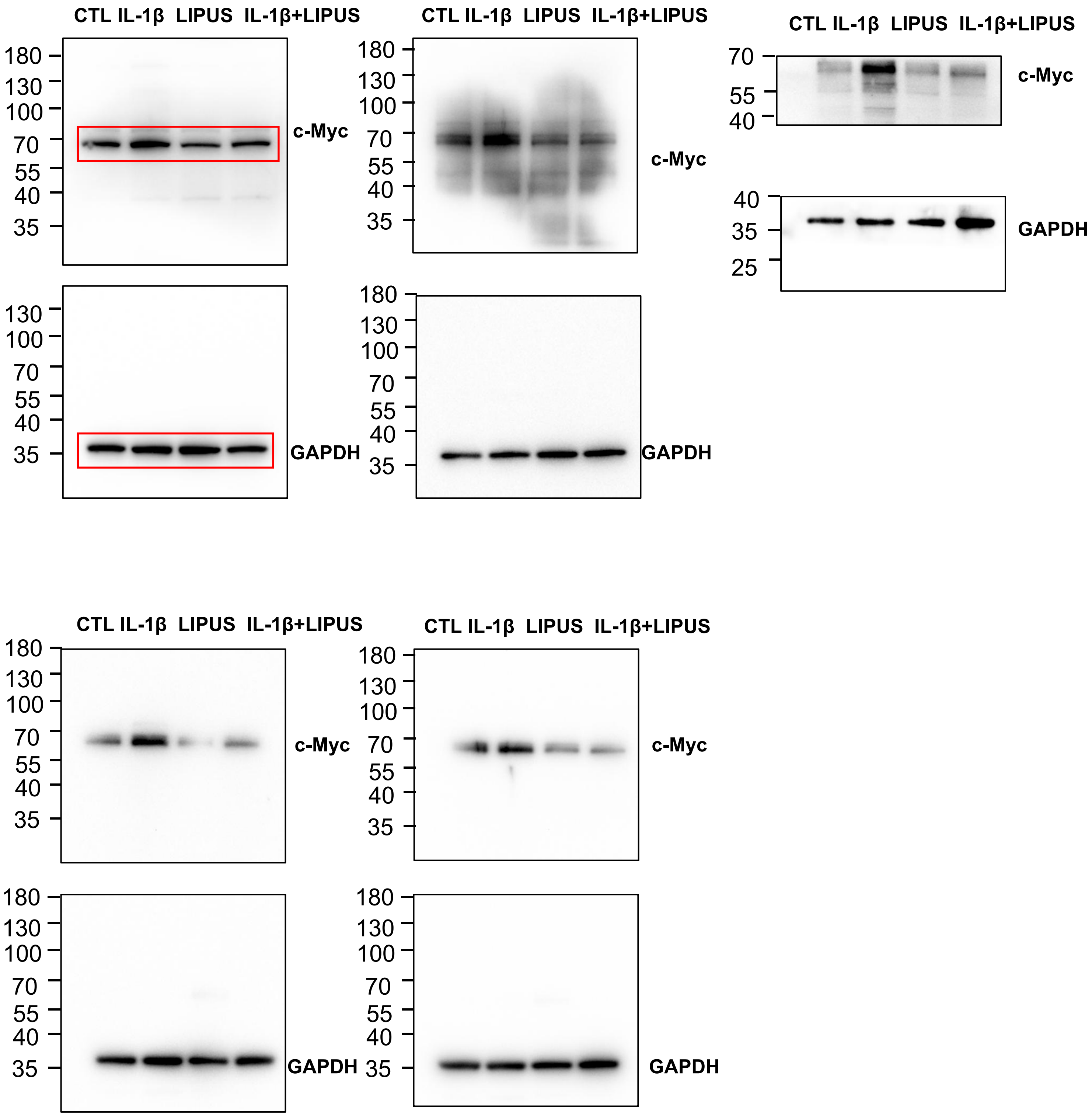

Figure 5D α-SMA & Fibronectin

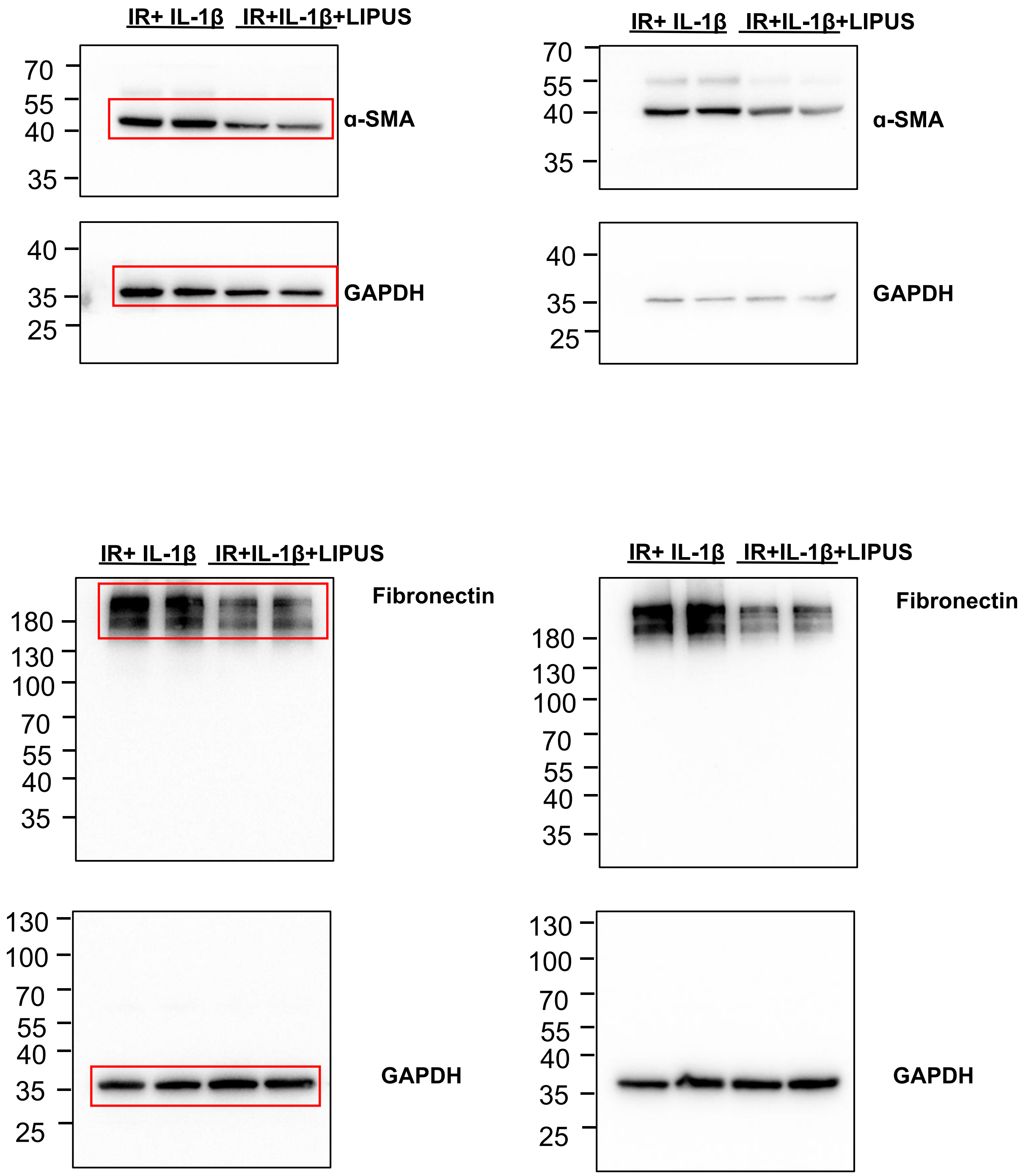

Figure 5E IL-1R & p-NF-κB & c-Myc

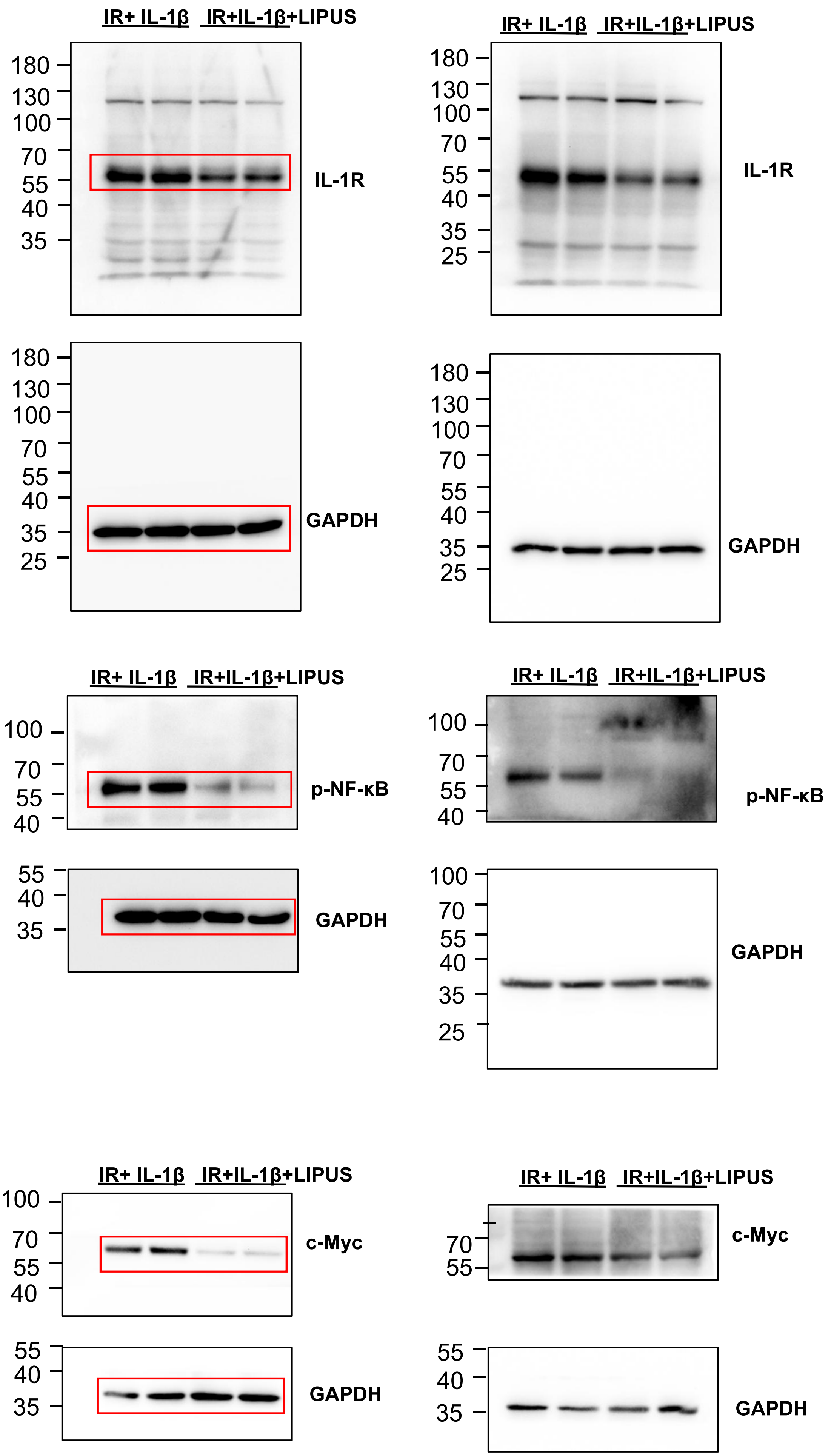

Figure 7D&7E α-SMA & c-Myc

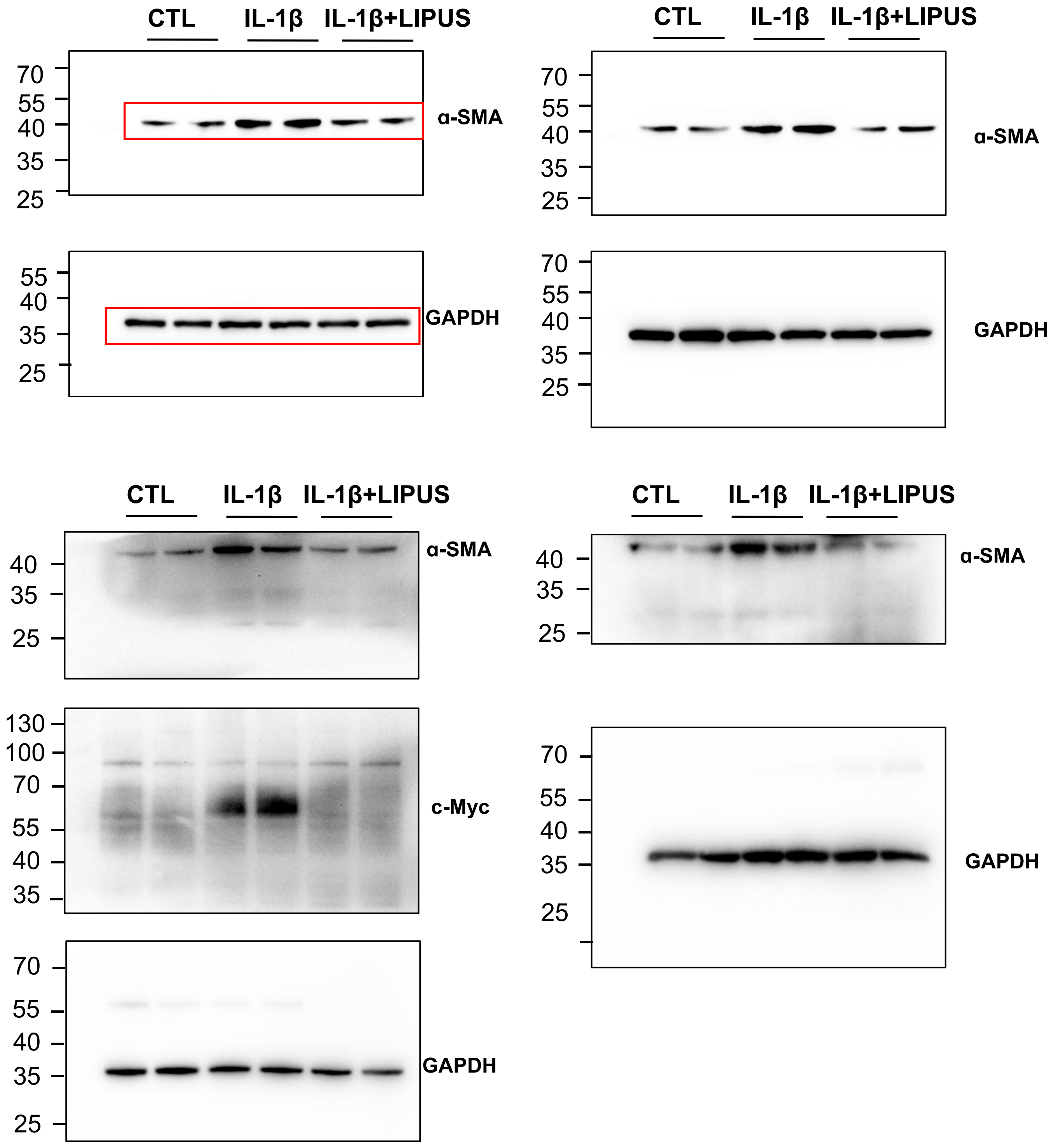

Figure 7D Fibronectin

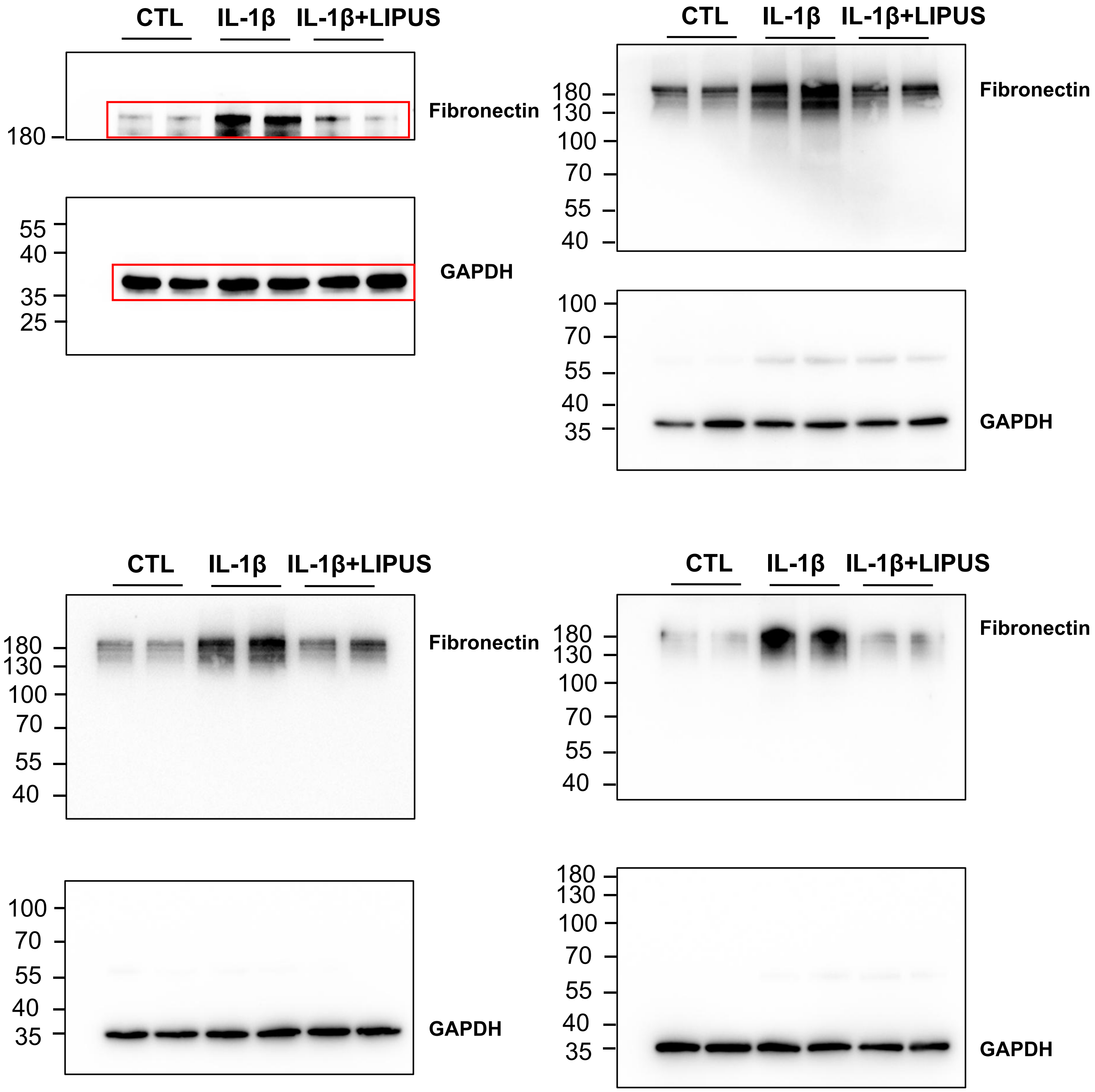

Figure 7E IL-1R

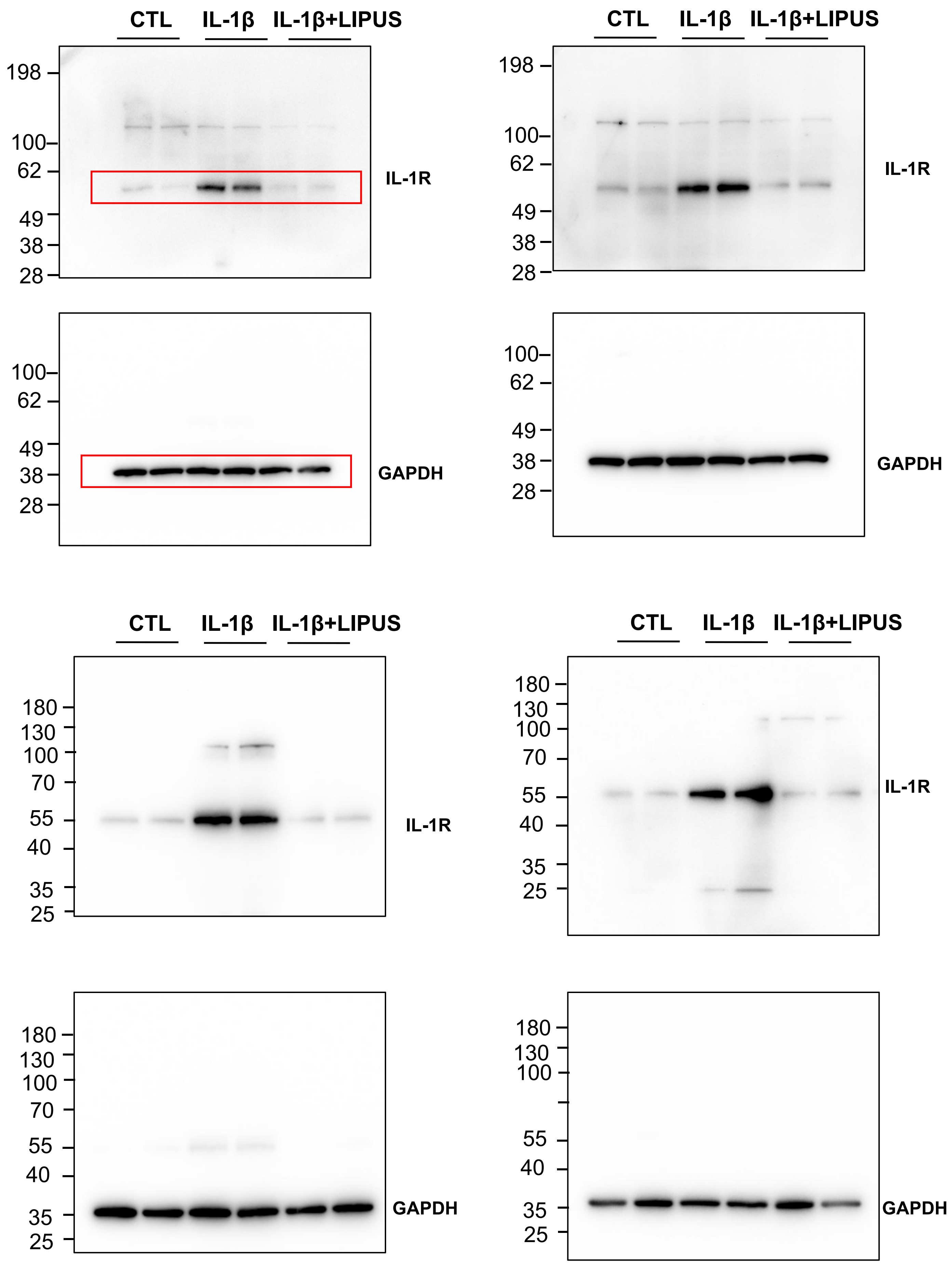

Figure 7E p-NF-κB

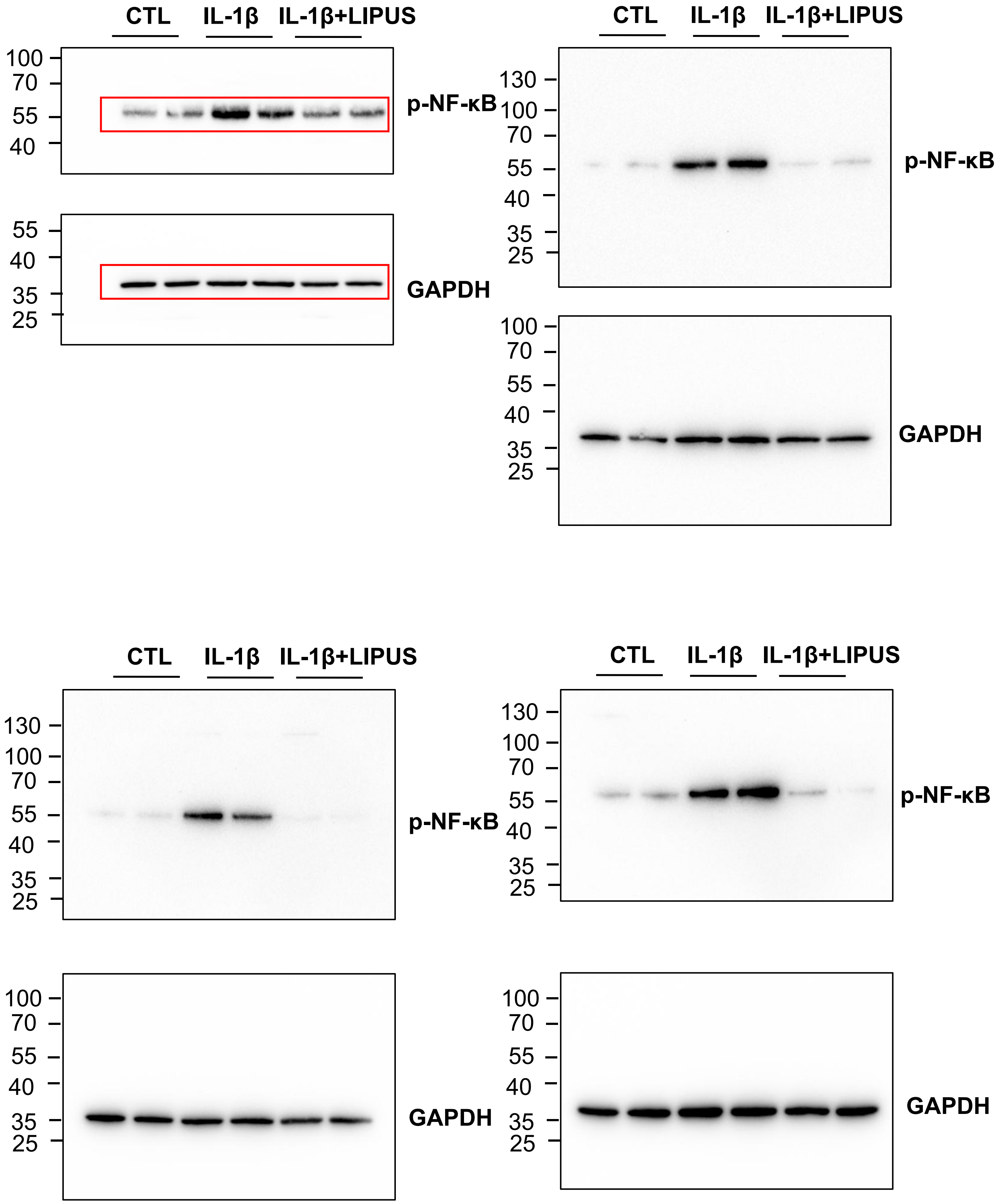

Figure 7E c-Myc

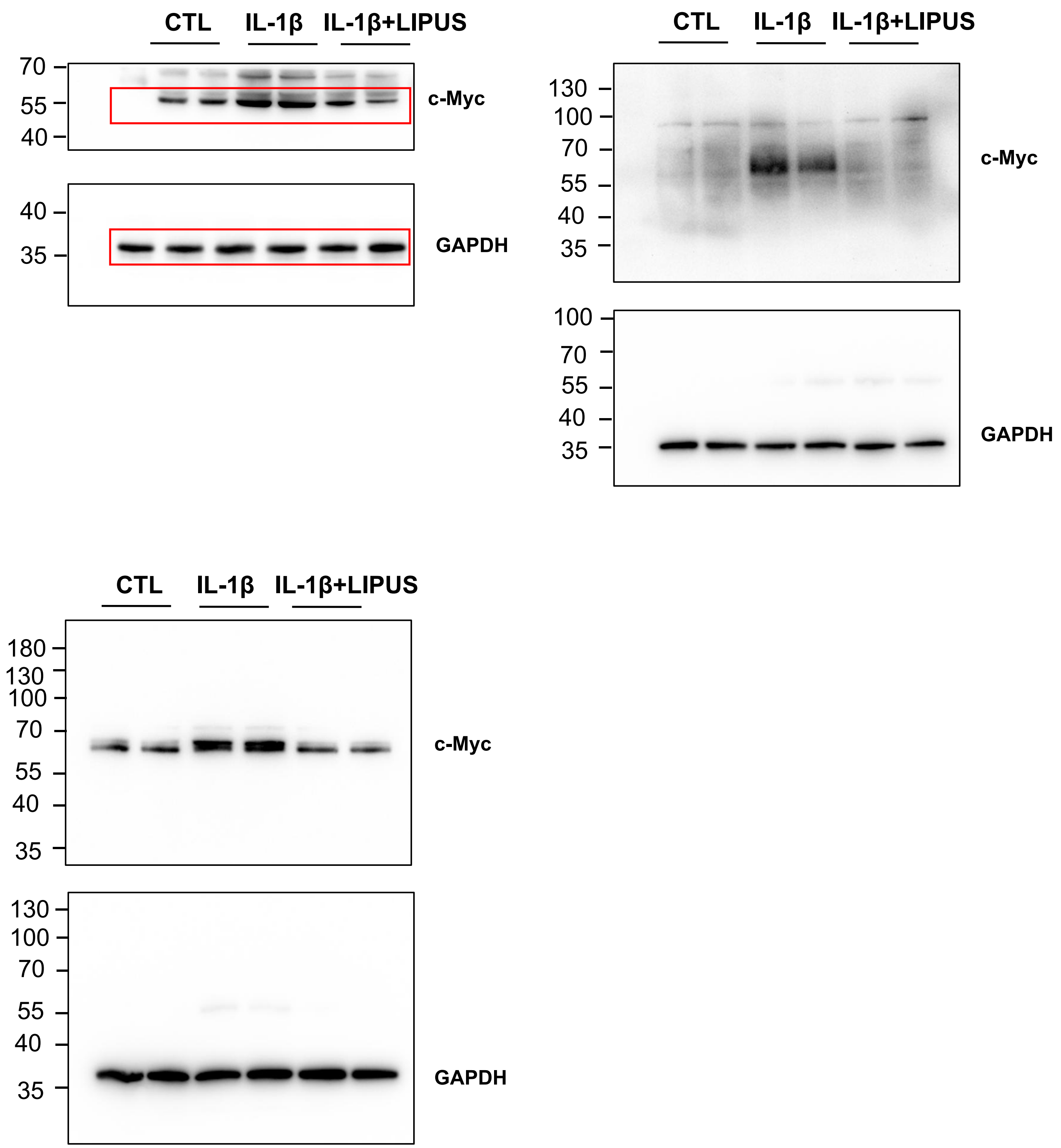

Figure 8D α-SMA & Fibronectin

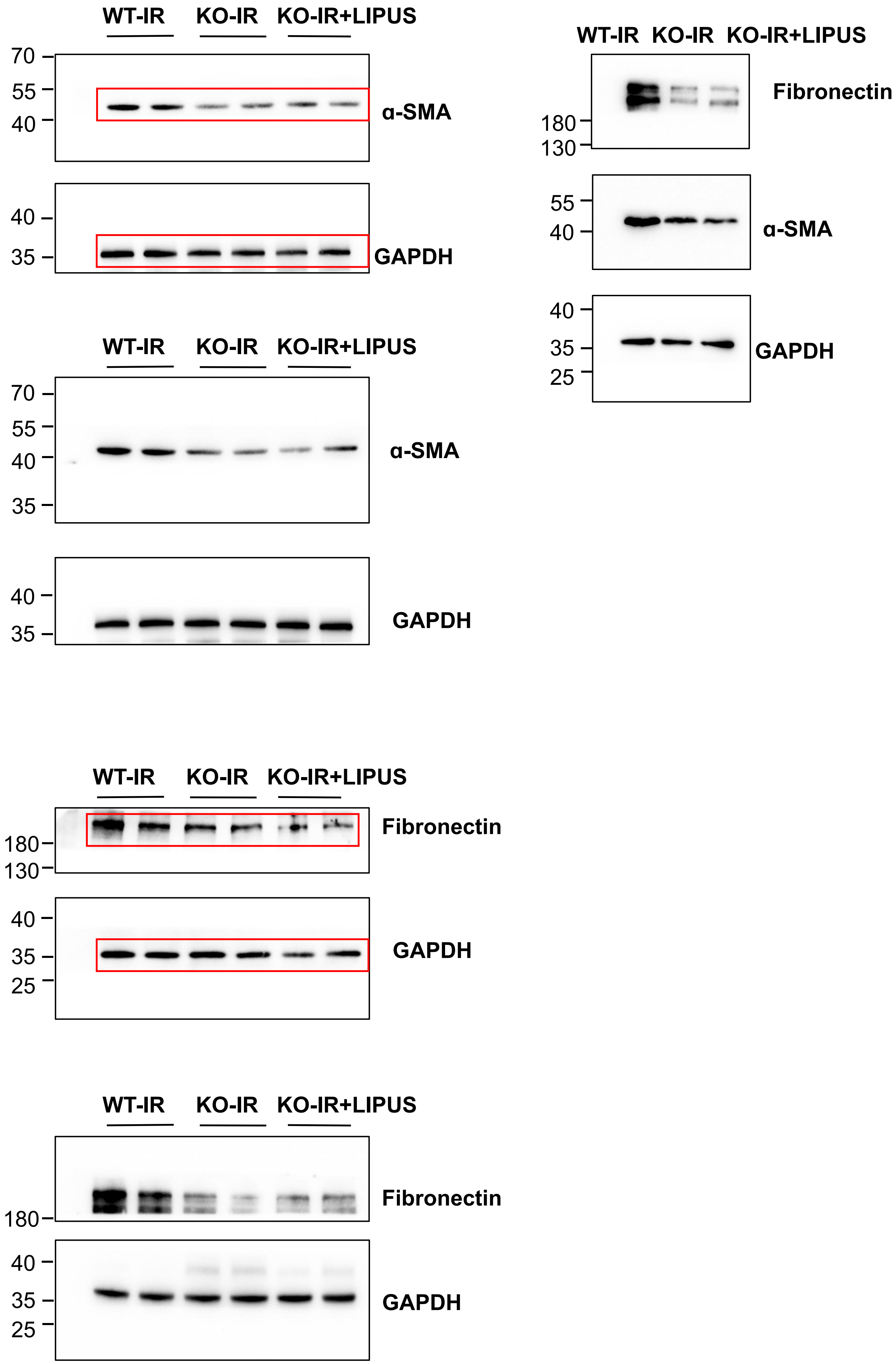

Figure 8D p-NF-κB & c-Myc

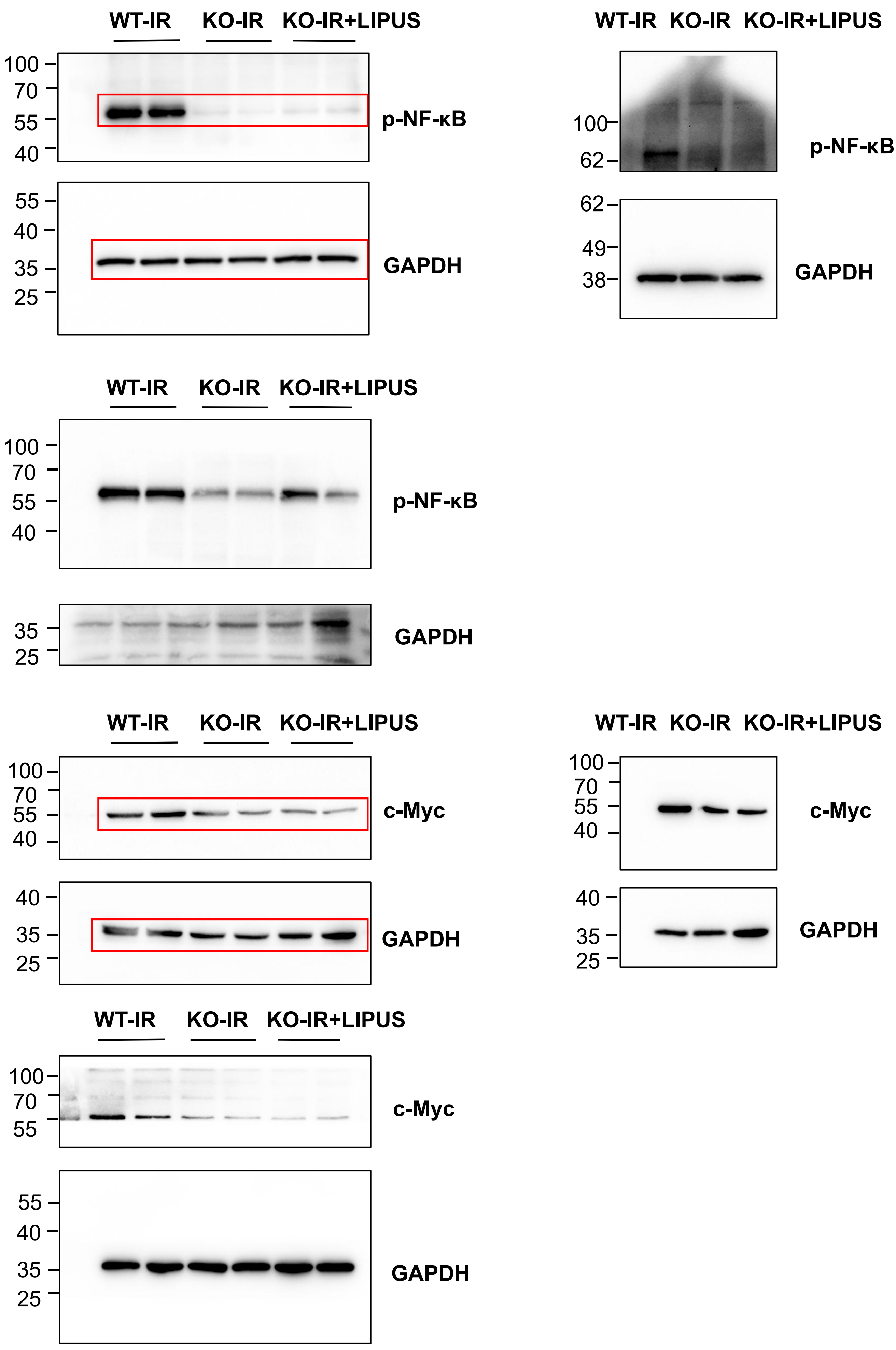

Supplement: Unedited blot and gel images [file jciinsight-10-186892-s022.pdf]
